# Stereoscopic optimization of industrial structure of the equipment manufacturing industry from the perspective of collaborative emissions reduction: Evidence from China

**DOI:** 10.1371/journal.pone.0232293

**Published:** 2020-04-30

**Authors:** Bin Qiao, Shunhang Jiang, Jiefei Zhang

**Affiliations:** College of Management and Economics, Taiyuan University of Science and Technology, Taiyuan, China; Institute for Advanced Sustainability Studies, GERMANY

## Abstract

Equipment manufacturing industry is one of the major industries of the Chinese economy. Previous researches have revealed that the industry has dilemmas of unreasonable industrial structure and high pollution. Using the data of 30 provinces in 2006-2015 in China, this study calculated a comprehensive pollution indicator when estimating the possible pollution reduction brought by the optimization of industrial structure and then evaluated the reasonable level of capital allocation of provinces and industries by using the methods of nonlinear programming and stochastic frontier method. Under the target of collaborative emission reduction, the results show that the optimized output of China’s equipment manufacturing industry could be increased by 5.42%, the energy intensity could be reduced by about 10.4%, and the comprehensive emission intensity could be reduced by about 7.47%. Due to the industry heterogeneity and regional heterogeneity, industrial capacity should be transferred between industries and regions. Since the capital investment in the equipment manufacturing industry is significantly mismatched between industries and regions, the capital allocation of provincial industries in China needs to be adjusted properly. This study provides theoretically and practically reference for collaborative pollution reduction, industry restructure, spatial layout and capital investment, which contributes to achieving the stereoscopic optimization of equipment manufacturing industry.

## 1 Introduction

Global environmental problems have become increasingly serious since the beginning of the 21st century. In 2018, global carbon emissions hit an all-time high of 33.14 billion tons, with an average emissions growth rate of approximately 1% to 2% [1]. Such problems have threatened not only human lives but also long-term prosperity. In line with the concept of “sustainable development,” governments of international communities have sought cooperation in dealing with environmental issues. At the 2014 APEC Summit in Beijing, China and the United States agreed to a “Joint Announcement on Climate Change,” in which China pledged to cap emissions by 2030. The Chinese government’s promotion of the restructuring and transformation of the country’s industrial development pattern seems clear. The equipment manufacturing industry, which is one of the core components of modern Chinese industries, provides production equipment for sectors across the national economy and has grown rapidly in recent years. In 2015, the equipment manufacturing industry accounted for 31.8% of the added value of the entire industry, and output value exceeded 20 trillion yuan [2]. Nonetheless, the energy consumption of China’s industries is higher than that of developed countries. In 2015, China’s energy intensity was approximately six times that of Japan and three times that of the United States [3]. On the other hand, the Chinese equipment manufacturing industry has experienced serious problems with the rising cost of productive factors, such as low production efficiency, overcapacity, and the serious waste of resources [4]. The traditional extensive development pattern of “high input, high consumption, and high emissions” has become unsustainable [5]. Targeting the problems of high pollution levels and overcapacity in the equipment manufacturing industry, “Made-in-China 2025” has proposed optimization of industrial structure and sustainable development as its main development goals. Achieving the Chinese equipment manufacturing industry’s goal to save energy, reduce emissions, and optimize industrial structure under the dual pressure of commitment to international emissions reduction cooperation and sustainable green development within the country is imperative.

As a capital-intensive industry, the Chinese equipment manufacturing industry has been facing the common problem of capital mismatch owing to the country’s unique capital structure and financial system. The low efficiency of capital allocation hinders the balanced development of industries nationwide [6]. In 2016, the Chinese equipment manufacturing industry lost 13.15% of its output owing to a mismatch of capital [7]. Thus, calculating the reasonable capital stock level for each region and industry is the key to promoting structural adjustment and balanced regional development. Overall, the resolution of a single problem is not enough to solve the current multiple predicament faced by the equipment manufacturing industry, such as high pollution, low production efficiency, overcapacity, and unreasonable industrial structure and spatial layout. The multi-aspect optimization of industrial structure, which is defined in this study as the stereoscopic optimization of industrial structure, should be analyzed. Stereoscopic optimization includes the following three meanings: 1) simultaneous achievement of collaborative emissions reduction and industrial structure optimization, 2) promotion of the rational input level of production factors and prevention of overcapacity, and 3) promotion of the rational allocation of capital among industries and regions and optimization of the industrial spatial layout.

In recent years, scholars have conducted various studies on sustainable industrial development [8–11]. Neoclassical growth theory holds a view that the extensive growth model dominated by factor expansion is unsustainable, and only intensive growth with increasing productivity can be sustained [12–15]. Several scholars have also examined the relationship between industrial structure optimization, energy saving, and emissions reduction. Fisher-Vanden et al. (2006) attributed the change of energy intensity to industrial restructuring, technological progress, and final demand [16]. Meanwhile, Kambara (1992) showed that reducing the scale of industries with high energy and pollution intensities while increasing the scale of industries with low energy and pollution intensities are essential in promoting industrial structure optimization and realizing energy saving and emissions reduction in the early stage of industrial evolution [17]. On the basis of this study, Zhu et al. (2014) used a multi-sector intertemporal dynamic optimization model to verify whether *CO*_2_ emissions can be reduced through the optimization of industrial structure and the potential value of energy saving and emissions reduction resulting from industrial structure optimization, as predicted by Mi et al. (2015) [18, 19]. To solve the problem of excess capacity and capital mismatch, Shi (2017) estimated the capacity utilization rate and the level of desirable capital in various industries by using the results of industrial structure optimization in the manufacturing industry [20]. Nonetheless, scholars have examined *CO*_2_ emissions as pollution indicators while ignoring the characteristics of China’s energy endowment and energy structure in its manufacturing industry. Coal consumption accounts for approximately 66% of Chinese total energy consumption. Compared with petroleum and other energy sources, coal combustion has a low calorific value and causes serious pollution, similar to *SO*_2_ and other pollutants, which are the main sources of air pollution in China [21]. Measure the true level of pollution in the manufacturing industry would be difficult if *CO*_2_ is taken as the sole source of pollution. Moreover, according to the “Environmental Air Quality Standards GB3095,” which was published by the China Environmental Protection Bureau in 1996 and 2012, *SO*_2_ is taken into consideration in air pollution indices AQI and API. In addition, given that capital allocation in the equipment manufacturing industry is unreasonable, previous studies have focused on capital mismatch among equipment manufacturing industries within the perspective of the nationwide industry according to industrial heterogeneity. This focus leads to the conclusion that a large amount of capital redundancy exists in the Chinese manufacturing industry [20]. However, most studies have ignored the regional heterogeneity and the interregional mismatch of capital [22], which could reduce the operability of the conclusions.

Based on the above analysis, collaborative emissions of pollutants and regional heterogeneity in capital allocation have considerable impacts on the industrial structure optimization of the Chinese equipment manufacturing industry. It is necessary to analyze the collaborative emission reduction from the perspective of provincial industries and calculate the reasonable capital allocation of each provincial industry. In short, the relevant researches on the capital investment and the relationship between the pollution reduction and the industrial structure adjustment can be summarized as follows: industrial structure adjustment reduces pollution emissions by reducing the energy intensity, and capital investment is significantly mismatched between industries and regions. Previous literatures provide a theoretical reference for the implementation of environmental protection policy and industry development planning. However, many scholars took *CO*_2_ as the only pollutant, which means ignoring other pollutants generated with energy combustion. In addition, the relevant researches only focus on national industries, lacking the analysis of inter-regional industrial adjustment combined with regional heterogeneity, and also ignore estimating the capital allocation of provincial industries.

Starting from the dilemmas in the equipment manufacturing industry, this study analyzes the realization of industrial structure optimization, reasonable allocation of capital and rational spatial layout of industries from the point of collaborative emission reduction, which contributes to the theory of industrial structure optimization. Compared with previous literatures, this study uses a comprehensive pollution indicator to take both *CO*_2_ and sulfur dioxide into consideration and then calculated the target value of industrial structure optimization. Theoretically, this study provides an analysis of industrial layout optimization and regional industrial transfer and gives a reasonable level of capital input in each provincial industry. Therefore, provincial panel data were used to make a more accurate analysis, which also helps to achieve energy conservation, emission reduction and industrial structure optimization. In order to realize the stereoscopic optimization of industrial structure, stochastic frontier regression and nonlinear programming based on environmentally friendly objective function are used.

The rest of this study is arranged as follows. The second section presents the literature review, and the third section introduces the methods of collaborative emissions reduction measurement, the industrial structure optimization model, and the settings of the related variables used in this study. The fourth section discusses the energy saving and emissions reduction, industrial structure adjustment, industry spatial shift, and rational capital allocation of the Chinese equipment manufacturing industry on the basis of the empirical results. The final section is the conclusion of the study.

## 2 Literature review

### 2.1 Industrial structure optimization and effect of collaborative emissions reduction

Optimization of the manufacturing industry structure and realization of emissions reduction have been hot topics in academic circles. Previous literatures have indicated that the economic development level, the industrial structure, energy intensity and investment level all impact pollution emissions [23–28]. Firstly, cutting down energy intensity is the key to breaking the coupling relationship between economic growth and pollution emissions [29]. Secondly, the evolution of industry structure is deeply affected by various factors such as trade and regional heterogeneity [30, 31]. The optimization and adjustment of industrial structure through policy guidance can significantly reduce energy intensity and thus reduce emission level [16, 32]. Many scholars have also reached similar conclusions through simulation analysis and STIRPAT (Stochastic by Regression on Population,Affluence,and Technology) [19, 32, 33].

Most of the above literatures only considers the optimization of the industrial structure at the carbon emission level but ignore the collaborative emission reduction effect brought by *CO*_2_ emission reduction. In fact, some scholars have applied the model of collaborative emission reduction that considers both *CO*_2_ and sulfur dioxide to verify this relationship [21, 34]. At the same time, given the heterogeneity of regional energy endowment and technological level [35], most of the relevant researches focus on national industries, ignoring regional heterogeneity in the energy-dependent equipment manufacturing industry, which will reduce the objectivity of the research conclusions. Through panel GMM method, input-output model and multi-objective planning, scholars have proved the necessity of making differentiated policies for different provinces and industries [36, 37].

Overcapacity and capital mismatch of the equipment manufacturing industry, as a capital-intensive industry, from the high capital investment has become hot topics among researchers in recent years. In fact, the key to solving these problems lies in the matching of production factors and optimized industrial structure. Due to the industrial and regional heterogeneity of capital mismatch in China’s equipment manufacturing industry [7], it is inevitable to study the capacity adjustment between industries and among regions, which also affects the spatial layout of provincial industries. Data envelope approach and other methods have been used to study the low utilization rate of manufacturing capacity [20], but regional and industrial heterogeneity have been ignored [6, 38–40].

In conclusion, the relationship between industrial structure optimization and pollution emission in the equipment manufacturing industry, as well as capital misallocation in the manufacturing industry have been studied in previous literature, which lays a foundation for the follow-up work, but there are still some limitations. Firstly, most studies used *CO*_2_ as the only source of pollution, ignoring the impact of sulfur dioxide, an important pollutant, on pollution emissions. Only by considering the emission reduction of major pollutants, namely *CO*_2_ and sulfur dioxide, can we identify the collaborative emission reduction effect and promote the green development of the industry. Secondly, most of the previous literatures conducted on the national industry, ignoring the regional heterogeneity of the industry and the differences in the energy structure and production technology between regions. The conclusion is not applicable to different provinces and regions. Thirdly, although some scholars have paid attention to the capital surplus caused by the capital-intensive characteristics of the equipment manufacturing industry, they have not analyzed the internal causes from the perspective of industrial structure optimization. Therefore, based on the previous research, we use the comprehensive pollution indicator considering *CO*_2_ and sulfur dioxide. Through the nonlinear programming method, the optimization value of the industrial structure of each province is obtained by taking the regional industry as the minimum decision-making unit, and then the reasonable capital allocation level of each province’s equipment manufacturing industry is calculated. Finally, the stereoscopic optimization of industrial structure including collaborative emission reduction, the rational layout of industries and reasonable allocation of capital can be realized in the equipment manufacturing industry.

## 3 Methodology

First, we must determine how to calculate the comprehensive pollution amount of *CO*_2_ and sulfur dioxide emissions. Second, we calculate the target value of industrial structure optimization. Finally, we analyze the matching of the capital level under the optimized value.

### 3.1 Estimation of comprehensive pollution coefficient

Kaya (1989) first put forward an equation to measure the level of carbon emissions in the manufacturing industry [41], as follows:

$$CE=\sum_{i}CE_{i}/E_{i}\times E_{i}/E\times E/Y\times Y/P\times P=\sum \delta \times \theta \times \rho \times \sigma \times P$$ (1)

where *i* represents the type of energy, and *CE*, *E*, *Y*, and *P* stand for emissions, energy consumption, provincial GDP, and population size of each province. The above indicators are transformed into corresponding coefficients of *δ*, *θ*, *ρ*, *σ*, representing the carbon emissions coefficient, energy structure, energy consumption per unit of output, and GNP per person, respectively. According to Eq (1), the *CO*_2_ coefficient is mainly affected by emissions, energy structure, energy efficiency, and other factors. Based on this finding, numerous scholars have attempted to measure the coefficient in different industries. By applying this equation along with the *CO*_2_ emissions coefficient published by the IPCC and NDRC, Shan et al. (2016) calculated a coefficient applicable to provincial manufacturing industries in China [42]. The present study uses the coefficient calculated by Shan et al. (2016) to measure the level of *CO*_2_ emissions in each region and industry [42].

Aside from *CO*_2_, *SO*_2_ and *NO*_*x*_ are also important sources of pollution in the equipment manufacturing industry [34]. Previous studies using a single carbon emission coefficient could not accurately measure the level of pollution in the equipment manufacturing industry, which made it difficult to achieve the goal of green industrial development. In this paper, on the basis of carbon emissions, *SO*_2_ is added to measure the pollution level of the manufacturing industry. On the one hand, coal combustion produces a large amount of *SO*_2_, and the coal-based energy structure is the main cause of air pollution and acid rain in China [43, 44]. Previously established knowledge has proved that the emission reduction of *SO*_2_ can save a lot of health costs [45]. In comparison, *NO*_*x*_ and *VOC* emission reductions bring relatively low health benefits [46], and emission reduction strategies for greenhouse gases (including *O*_3_, *N*_2_ *O*, etc.) cannot guarantee net health benefits [47]. In addition, empirical evidence from China and Malaysia shows that the reduction of one pollutant will lead to the reduction of others [34, 48]. Therefore, compared with other pollutants, the control of *SO*_2_ has a more far-reaching impact on China’s green development, and the emission of other pollutants behind the reduction of *SO*_2_ will also be controlled to a certain extent. The present study extends *SO*_2_ emissions based on Eq (1) as Eq (2).

$$\text{S}{\text{E}}_{\text{k}}=\sum_{\text{i}}\text{S}{\text{E}}_{\text{jk}}/\text{C}{\text{E}}_{\text{jk}}\times \text{C}{\text{E}}_{\text{jk}}(1/{\text{E}}_{\text{jk}}\times {\text{E}}_{\text{jk}}\text{/}{\text{E}}_{\text{k}}\times {\text{E}}_{\text{k}}\text{/}{\text{Y}}_{\text{k}}\times {\text{Y}}_{\text{k}}/{\text{P}}_{\text{k}}\times {\text{P}}_{\text{k}})$$ (2)

On the basis of the above decomposition of carbon emissions, Eq (2) extends *SE*, that is, emissions. *J* represents the type of energy, *K* represents provinces or regions, and represents sulfur dioxide emissions per unit of emissions, that is, the comprehensive pollution coefficient. This coefficient exhibits strong heterogeneity because it is affected by factors such as production technology, energy endowment, and the intensity of environmental regulation. Therefore, it can affect the industrial pollution level as well as the industrial production technology level and regional energy structure. Data on Chinese provinces from 2006 to 2015 are used, and the results from calculating the comprehensive pollution index of each province are shown in Table 1.

**Table 1:** Comprehensive pollution coefficient of provinces and regions in China.

Provinces	Comprehensive Pollution Coefficient	Provinces	Comprehensive	Provinces	Comprehensive Pollution Coefficient
Anhui	0.0065397	Heilongjiang	0.0081561	Shandong	0.0106381
Beijing	0.0077434	Hubei	0.0090777	Shanxi	0.0085837
Fujian	0.0111489	Hunan	0.0082635	Shaanxi	0.0092136
Gansu	0.0060927	Jilin	0.0077018	Shanghai	0.0101126
Guangdong	0.0088554	Jiangsu	0.0089765	Sichuan	0.0083340
Guangxi Zhuang Autonomous Region	0.0088554	Inner Mongolia Autonomous Region	0.0068151	Xinjiang Uygur Autonomous Region	0.0075850
Guizhou	0.0084229	Liaoning	0.0094489	Tianjin	0.0074553
Hainan	0.0042271	Jiangxi	0.0093043	Yunnan	0.0088554
Hebei	0.0122997	Ningxia Hui Autonomous Region	0.0086284	Zhejiang	0.0093646
Henan	0.0122997	Qinghai	0.0086284	Chongqing	0.0036922

The comprehensive pollution emissions *S* for each subindustry can be calculated according to the comprehensive pollution coefficient above. The calculated comprehensive emissions *S* represents not only the amount of *SO*_2_ pollution but also carbon emissions and various factors affecting *CO*_2_ emissions. Therefore, this study takes *S* as a constraint in the follow-up industrial structure optimization model.

### 3.2 Industrial structure optimization of the equipment manufacturing industry

In this study, the output structure of the equipment manufacturing industry represents industrial structure, and the nonlinear programming model based on the function of environmental intensity is used as the optimization model of the industrial structure of the equipment manufacturing industry. The nonlinear programming model mainly considers the adjustment of output structure, comprehensive pollution, and so on, which are represented by output *Y*, employee *L*, energy consumption *E*, and pollution emissions *S*. The model is set as follows:

$$\begin{array}{c} MinT{P_{t}}^{*}=\gamma_{EP}\cdot E{P_{t}}^{*}+\gamma_{SP}\cdot S{P_{t}}^{*}\\ s.t{Y_{i,j,t}}^{*}\cdot EP_{i,j,t}={E_{i,j,t}}^{*}\\ {Y_{i,j,t}}^{*}\cdot SP_{i,j,t}={S_{i,j,t}}^{*}\\ {Y_{i,j,t}}^{*}\cdot LP_{i,j,t}={L_{i,j,t}}^{*}\\ -\left(1+\theta_{i,t}\right)IM_{i,t}\leq \sum_{j=1}^{R}\left({Y_{i,j,t}}^{*}\right)-\sum_{k=1}^{m+n}\left(1+\mu_{i,t}\right)\alpha_{i,k,t}{Y_{k,t}}^{*}-\left(1+\psi_{i,t}\right)XF_{i,t}\leq \left(1+\delta_{i,t}\right)EX_{i,t}\\ \sum_{i}^{m}\sum_{j=1}^{R}({E_{i,j,t}}^{*})\leq \sum_{i}^{m}\sum_{j=1}^{R}E_{i,j,t}\\ \sum_{i}^{m}\sum_{j=1}^{R}({S_{i,j,t}}^{*})\leq \sum_{i}^{m}\sum_{j=1}^{R}S_{i,j,t}\\ \left(1-\lambda_{j,t}\right)\sum_{i}^{m}L_{i,j,t}\leq \sum_{i}^{m}\left({L_{i,j,t}}^{*}\right)\leq \left(1+\lambda_{j,t}\right)\sum_{i}^{m}L_{i,j,t}\\ EP_{t}^{*}=\sum_{i}^{m}\sum_{j=1}^{R}(E_{i,j,t}^{*})/\sum_{i}^{m}\sum_{j=1}^{R}(Y_{i,j,t}^{*})\\ SP_{t}^{*}=\sum_{i}^{m}\sum_{j=1}^{R}(S_{i,j,t}^{*})/\sum_{i}^{m}\sum_{j=1}^{R}(Y_{i,j,t}^{*}) \end{array}$$ (3)

Where *i*(*i* = 1, 2, 3…*M*), *k*(*k* = 1, 2, 3…*N*), *j*(*j* = 1, 2, 3…*R*) and *t* indicate the subsectors of the equipment manufacturing industry, other industries except the equipment manufacturing industry, provinces, and years, respectively; *TP*, *EP*, and *SP*, represent the environmental intensity, energy intensity, and pollution intensity of the equipment manufacturing industry in t year, respectively; *Y*, *EP*, *E*, *SP*, *S*, *LP*, and *L* indicate output, energy intensity, energy consumption, pollution intensity, pollution emissions, employment intensity, and employment, respectively; *γ*_*EP*_ and *γ*_*SP*_ represent the proportion of energy intensity and sulfur dioxide intensity, respectively; *θ*, *α*, *μ*, *ψ*, *δ* and λ represent the import change rate, the direct consumption coefficient, the direct consumption coefficient change rate, other consumption change rates, the export change rate, and the employment change rate, respectively.

The objective function of nonlinear programming is to minimize the intensity of resources and emissions. According to this research idea, the constraint function mainly includes the following contents.

First, the existing technical level, energy structure, and employee structure of the Chinese equipment manufacturing industry will not change substantially in a certain period. Therefore, this study calculates the energy intensity, pollution intensity, and employment intensity of the equipment manufacturing industry in various provinces and regions according to the 2015 data. The optimized output *Y*, energy consumption *E*, pollution emissions *S*, and employment *L* are constrained according to the relevant intensity, thereby providing a reference for the reasonable adjustment of the spatial layout of the equipment manufacturing industry across industries and regions.

Second, because the output of the manufacturing industry is closely related to the fluctuation of supply and demand in an open economy, placing a restriction on the output of various industries is necessary. The lower limit of the output restriction should meet the input of intermediate products in other industries as well as residents’ and the government’s consumption when considering the import. In addition, the upper bound is the output minus the export, while satisfying the consumption and intermediate product input of other industries.

Third, given that the smallest decision-making unit in linear programming is provincial industries, ensuring that the employed population in each province or region is still within a reasonable range is important. In this study, the absence of a large gap in the labor force, mass unemployment, or labor migration is assumed. Therefore, the average change rate of the labor force in the sample period is taken to calculate the upper and lower bounds of the total employed population in each province.

Fourth, the total energy consumption and total emissions *S* of the equipment manufacturing industry are restrained as a whole.

### 3.3 Matching of capital input

On the basis of the above analysis results of industrial structure optimization, this study further uses the historical data of provincial industries to measure the nonlinear relationship between the input and output of the equipment manufacturing industry and calculates the capital input level matching the optimal output scale according to the existing production input and factor allocation of provinces, regions, and industries.

This study establishes a production function in the form of transcendence of logarithm on the basis of the stochastic frontier analysis model of Battese and Coelli (1995) [49]. A stochastic frontier estimation method is used to estimate the model to better control the disturbance of random error terms. This procedure is conducive to the objective estimation of the nonlinear relationship between the input and output of production factors. Factors of production include capital *K*, labor *L*, intermediate product input *M*, and technical level *T*. The quadratic terms of each factor and interactive term are added to the estimation model to properly describe the nonlinear relationship between the input and output. The specific settings of the model are as follows:

$$\begin{array}{cc} LogY_{it} & =\beta_{0}+\beta_{1}LogK{}_{it}+\beta_{2}LogL_{it}+\beta_{3}LogM_{it}+\beta_{4}Log{K_{it}}^{2}+\beta_{5}Log{L_{it}}^{2}+\beta_{6}Log{M_{it}}^{2}\\ & \;+\beta_{7}LogK{}_{it}LogL_{it}+\beta_{8}LogK{}_{it}LogM_{it}+\beta_{9}LogL_{it}LogM_{it}+\beta_{10}T_{t}+\beta_{11}{T_{t}}^{2}\\ & \;+\beta_{12}T_{t}LogK{}_{it}+\beta_{13}T_{t}LogL_{it}+\beta_{14}T_{t}LogM_{it}+V_{it}-U_{it} \end{array}$$

### 3.4 Data source and variable definition

According to China’s national industrial classification, GBT475, which was published in 2017, the equipment manufacturing industry includes (1) the metal products industry, (2) the ordinary machinery industry, (3) the equipment for special purposes industry, (4) the transportation equipment industry, (5) the electric equipment and machinery industry, (6) the electronics and telecommunications equipment industry, and (7) the instruments, meters, cultural, and office machinery industry. Given that significant differences exist in the statistical coverage of statistical yearbooks in different provinces and regions, numerous missing values in the energy consumption and pollution data exist in each subindustry of 30 provinces after 2015. And due to the lack of data on Xizang, Hong Kong, Macao and Taiwan, this study uses the data of 30 provinces and regions in China from 2006 to 2015 for research, which is similar to the method of data selection in relevant literatures [50, 51]. The definitions and sources of the variables are shown in Table 2.

**Table 2:** Data source and variable definition.

Variable name	Variable definition	Data sources
Output Y	Gross Industrial Output	Statistical Yearbook
Energy consumption E	Calculation based on apparent accounting method of fossil energy consumption scale	Statistical Yearbook
Comprehensive emissions S	Based on IPCC and NDRC carbon dioxide emission coefficient and comprehensive pollution coefficient	Statistical Yearbook
Employment L	Employment at the end of the year	Statistical Yearbook
Capital investment K	Perpetual inventory method	Statistical Yearbook
Intermediate Product Input M	*M* = *Y*(−*inc* − *tax*)/*P*_*t*_	Statistical Yearbook
Technical level T	Represented by time scale	
Energy intensity EP	Energy Consumption/Gross Industrial Output	
Comprehensive Emission Intensity SP	Comprehensive Emissions/Gross Industrial Output	
Employment intensity LP	Employment/gross industrial output	
Import and export volume IM,EX	Import and export volume of industries	Statistical Yearbook
Other consumption XF	Data from tables	China’sInput- Output Table
Employment change rate λ	Employment Change Rate in that Year	National Bureau of Statistics
Import and export rate of change *θ*, *δ*	Change rate of import and export in that year	National Bureau of Statistics
Energy Intensity Coefficient *γ*_*EP*_	Take 0.5	
Emission Intensity Coefficient *γ*_*SP*_	Take 0.5	

The apparent emissions accounting approach [42] represents apparent fossil fuel consumption = local production + import–export + transfer-out provinces + inventory changes—non-energy use loss. The comprehensive emissions *S* for each provincial subdivision industry is calculated based on this equation. The energy intensity and emissions intensity coefficients are set as 0.5, because the settings of these two variables have little influence on the results.

## 4 Results and discussions

### 4.1 Preliminary relations between pollution level, output, and factor input in the equipment manufacturing industry

Given the continuous expansion of the output scale of the Chinese equipment manufacturing industry, problems in environmental pollution and overcapacity have prevailed. The development level of the equipment manufacturing industry in different industries and provinces has varied substantially for a long time, which has resulted in a marked regional imbalance.

**Fig 1:**
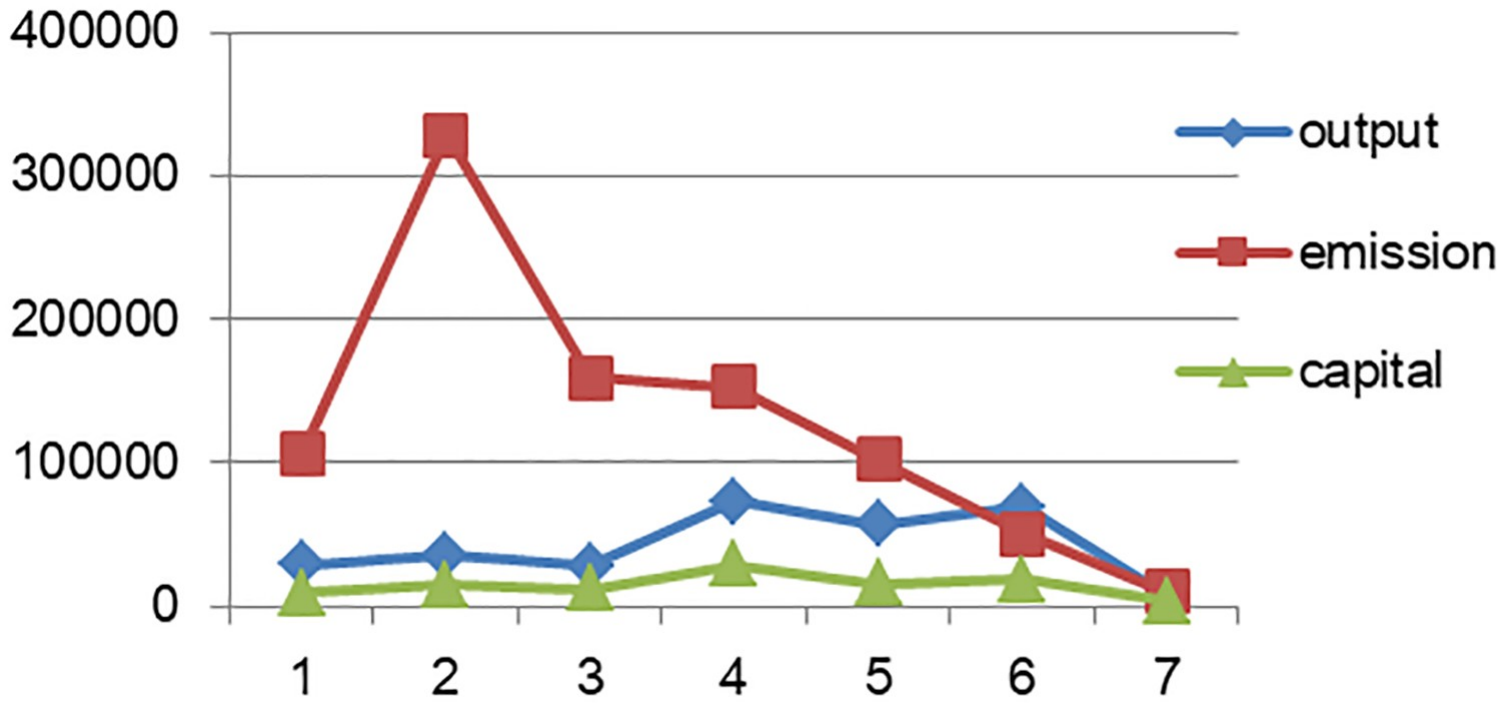
Output, pollution, and capital allocation of equipment manufacturing industries.

**Fig 2:**
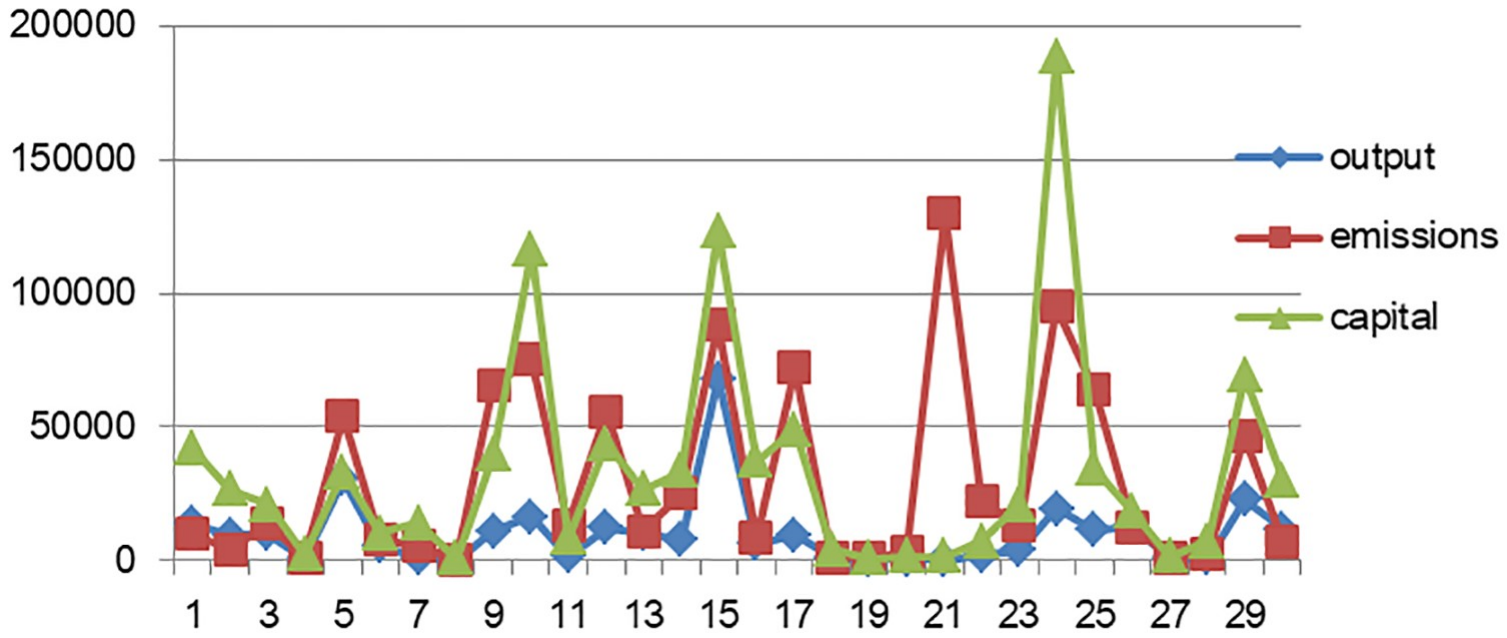
Output, pollution, and capital allocation of provinces and other areas.

Figs 1 and 2 show that correlations exist between total output, pollution intensity, and the level of capital allocation. The development level of these variables likewise vary considerably across industries, provinces, and regions.

The descriptive result from Table 3 further shows that the output scale of provincial equipment manufacturing differs remarkably. Moreover, the emissions intensity and labor intensity between provinces and regions vary substantially, thereby indicating that the development status, employment levels, and production technology of the industries in different provinces and regions are quite different.

**Table 3:** Descriptive statistics of output, emissions intensity, and labor intensity.

	Industry	Max	Min	Mean	Variance
Output	Metal Products	5976.97	11.66	948.15	1581436.28
	Ordinary Machinery	8498.98	0.19	1191.80	2829660.35
	Equipment for Special Purposes	5705.82	1.21	938.17	1310898.80
	Transportation Equipment	10488.06	2.99	2423.20	6342783.22
	Electric Equipment and Machinery	15673.19	33.05	1905.03	9800230.63
	Electronic and Telecommunications Equipment	18207.88	1.59	2388.90	16471257.95
	Instruments, Meters, Cultural and Office Machinery	3299.00	0.85	237.65	349953.02
Emission Intensity	Metal Products	740.59	0.39	30.49	17442.54
	Ordinary Machinery	2981.16	0.96	120.48	287704.15
	Equipment for Special Purposes	3519.98	0.30	122.44	398096.21
	Transportation Equipment	3101.69	0.10	106.21	309419.54
	Electric Equipment and Machinery	334.00	0.20	13.81	3560.38
	Electronic and Telecommunications Equipment	1111.97	0.05	39.90	41052.64
	Instruments, Meters, Cultural and Office Machinery	410.09	0.08	24.37	6334.25
Labour Intensity	Metal Products	952703.70	995.82	37009.63	28936967137
	Ordinary Machinery	1556686.83	596.25	83944.54	96735090852
	Equipment for Special Purposes	3540089.39	878.48	131522.90	402524040419
	Transportation Equipment	1585056.96	152.30	60159.28	80582661205
	Electric Equipment and Machinery	211492.44	274.31	9759.35	1406862294
	Electronic and Telecommunications Equipment	3084135.33	326.89	121141.37	315149817023
	Instruments, Meters, Cultural and Office Machinery	4416161.68	1804.18	199081.46	620111289313

This finding suggests that regional heterogeneity and interindustry heterogeneity should not be neglected in the study of industrial structure optimization of the equipment manufacturing industry. Moreover, this heterogeneity also provides room for the rationalization of the spatial layout of industrial structure at the national level.

### 4.2 Analysis of output scale, pollution level, and capital allocation of the equipment manufacturing industry

In this study, we use a nonlinear programming method to calculate the target value of industrial structure optimization. Along with minimum energy consumption intensity and emissions intensity, we obtain the total output of the equipment manufacturing industry and the optimal distribution of industries in each province in 2015 and further estimate the capital allocation level of industries for each province.

The total environmental intensity index of 30 provinces in China can be reduced from 2107.19 to 1932.68, and the overall intensity can be reduced by approximately 8.28% through optimization of industrial structure and distribution adjustment in different provinces. Energy intensity is expected to drop by approximately 10.4%, emissions intensity can be reduced by approximately 7.47%, overall energy consumption can be cut by approximately 5.54%, and collaborative emissions reduction is estimated to be reduced by approximately 2.45%. Under the constraint of collaborative emissions reduction, the overall output of the equipment manufacturing industry can be increased by 5.42%, and the total output value is expected to increase from 29,959.78 billion yuan to 31,478.4 billion yuan. Jobs could also be provided with the same total amount. According to the Annual Report on China’s Low-carbon Economic Development, which was published in 2017, the country’s carbon emissions decreased by approximately 0.6% in 2015. Based on the idea of systematic optimization of industrial structure, optimization could theoretically achieve four times the effect of actual emissions reduction, which proves the necessity and effectiveness of stereoscopic optimization of industrial structure. This result is similar to Li’s (2017) conclusion that industrial structure optimization can effectively reduce carbon emissions. Furthermore, this paper analyzes the provincial industries and takes sulfur dioxide into consideration, which indicates the existence of collaborative emission reduction [33].

### 4.3 Analysis of collaborative emissions reduction

According to the results of the aforementioned collaborative emissions reduction optimization, the pollution level of the Chinese equipment manufacturing industry can be reduced by approximately 2.454% on average. The pollution level of different industries has changed owing to the difference in natural endowment and production technology. Specifically, only the ordinary machinery and electronics and telecommunications equipment industries have slightly increased pollution levels among the seven major industries, whereas the pollution levels in the remaining five industries have substantially decreased (Table 4).

**Table 4:** Proportion of collaborative emissions reduction in the equipment manufacturing subdivisions.

Subdivision industry	Original value	After optimization	Emission reduction ratio
Metal Products	10.55	9.89	0.0627
Ordinary Machinery	32.90	33.30	-0.0120
Equipment for Special Purposes	15.98	15.38	0.0373
Transportation Equipment	15.24	13.98	0.0823
Electric Equipment and Machinery	10.18	9.950	0.0224
Electronic and Telecommunications Equipment	5.06	5.19	-0.0264
Instruments, Meters, Cultural and Office Machinery	1.03	1.01	0.0229
Gross value	90.93	88.70	0.0245

The overall pollution intensity of the Chinese equipment manufacturing industry is predicted to decrease from 3.05 to 2.82 (Table 5) through the changes in the industries. The output pollution intensities of other industries have decreased markedly except for the ordinary machinery industry and the equipment for special purposes industry, which proves the considerable emissions reduction effect of systematic optimization of industrial structure.

**Table 5:** Change in pollution intensity in the output of various industries.

Pollution Intensity in Output	Original value	After optimization	Reduced proportions
Metal Products	3.71	3.66	0.015
Ordinary Machinery	9.20	9.20	0.001
Equipment for Special Purposes	5.68	5.68	-0.001
Transportation Equipment	2.10	1.98	0.056
Electric Equipment and Machinery	1.78	1.66	0.069
Electronic and Telecommunications Equipment	0.73	0.66	0.092
Instruments, Meters, Cultural and Office Machinery	1.44	0.65	0.552
Gross value	3.05	2.82	0.075

#### 4.3.1 Discussion on the effect of collaborative emissions reduction

In contrast to previous studies [18], this study uses non-single pollutants to calculate industrial pollution levels in different regions. To verify the existence of the collaborative emissions reduction effect in this study, we firstly try to take *CO*_2_ emissions as the only pollutant in the optimization equation for comparative analysis. The optimization results show that carbon emissions are expected to drop by only 1.79%. Secondly, according to the results of the previous optimization, this study uses a comprehensive pollution index to calculate total *CO*_2_ emissions reversely and finds that optimization could reduce carbon emissions by 2.452% (Table 6). This finding further proves the existence of the collaborative emissions reduction effect and verifies the aforementioned theoretical expectations. Analysis of the collaborative emissions reduction effect can better describe the pollution emissions situation for each province and provide a practical reference for industrial structure optimization under the requirements of green development, which also promotes the realization of stereoscopic optimization of industrial structure of the equipment manufacturing industry. Fu et al. (2017) verified the existence of a collaborative emission reduction effect by using sample data of China’s power industry [34]. This study draws similar conclusions using data from different industries.

**Table 6:** Calculated changes in carbon emissions.

Carbon emissions	Original value	After optimization	Reduced proportions
Metal Products	10.59	11.38	0.0687
Ordinary Machinery	34.46	34.02	-0.0129
Equipment for Special Purposes	15.97	16.64	0.0399
Transportation Equipment	15.78	17.01	0.0724
Electric Equipment and Machinery	10.39	10.61	0.0212
Electronic and Telecommunications Equipment	5.46	5.31	-0.0280
Instruments, Meters, Cultural and Office Machinery	1.11	1.15	0.0358
Gross value	96.13	93.77	0.0245

### 4.4 Analysis of stereoscopic optimization of industrial structure in the equipment manufacturing industry

#### 4.4.1 Output change from the perspective of industries

The total output value of the equipment manufacturing industry has increased significantly, while the heterogeneity of the industry is apparent. Under the condition of satisfying the consumption of residents and the government and the supply of intermediate products, the total output values of the metal products industry, the equipment for special purposes industry, and the transportation equipment industry have declined to a certain extent, with rates of 4.87%, 3.78%, and 2.83%, respectively.

**Fig 3:**
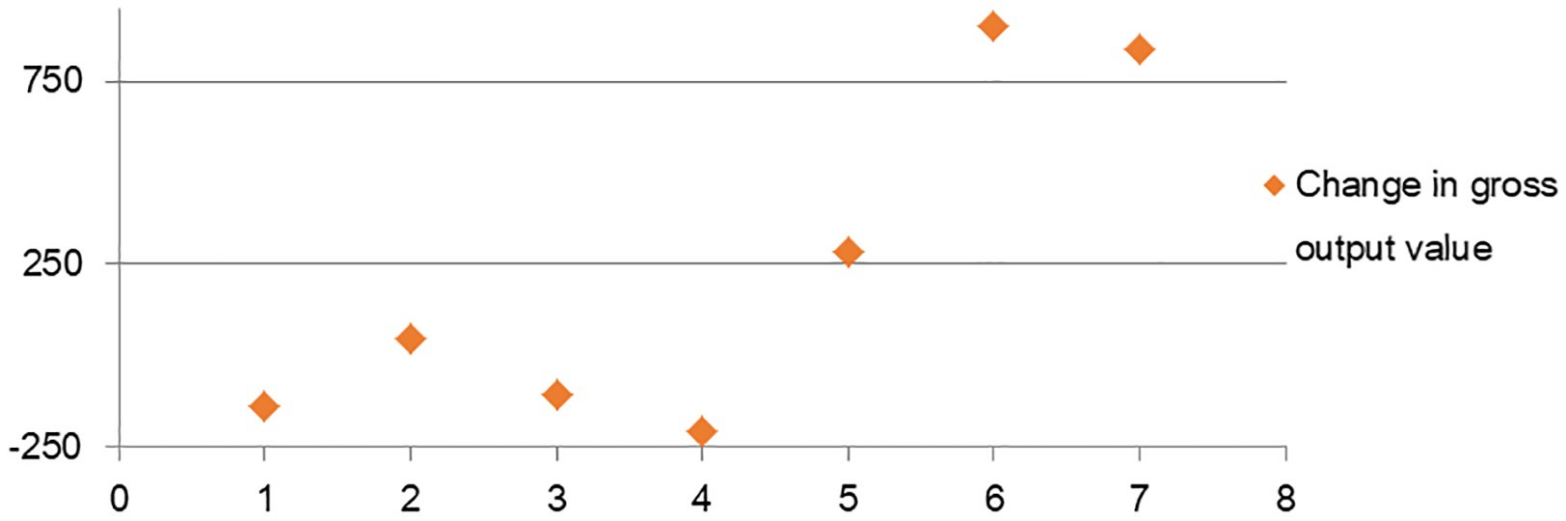
Changes in output value after optimization of industrial structure.

The total output values of the ordinary machinery, electric equipment and machinery, electronics and telecommunications equipment, and instruments, meters, cultural, and office machinery industries have significantly increased. Among them, the electronics and telecommunications equipment and instruments, meters, cultural, and office machinery industries have improved most significantly, with increases in their total output values by approximately 900 and 840.5 billion yuan (Fig 3).

Fig 3 shows that industries with relatively high pollution intensity and low technology intensity have reduced production in varying degrees, such as the metal products and transportation equipment industries and so on. High-technology industries with relatively low pollution intensity, such as the electronics and telecommunications equipment and instruments manufacturing industries, have significantly increased output scales. Obviously, by optimizing the industrial structure of various industries in the equipment manufacturing industry, rationalization and supererogation of industrial structure can be promoted, which also contributes to the stereoscopic optimization. The conclusion of this paper is consistent with that of basic research in related fields [27, 28], verifying the potential of energy conservation and emission reduction brought about by industrial restructuring.

#### 4.4.2 Industrial adjustment from the perspective of provinces and regions

The constraints of labor intensity and employment volume are added in the nonlinear programming model to ensure employment and prevent population congestion. The output value of the equipment manufacturing industry in different provinces has changed considerably under the influence of the objective function, which can result in the optimization of the industrial spatial pattern.

**Fig 4:**
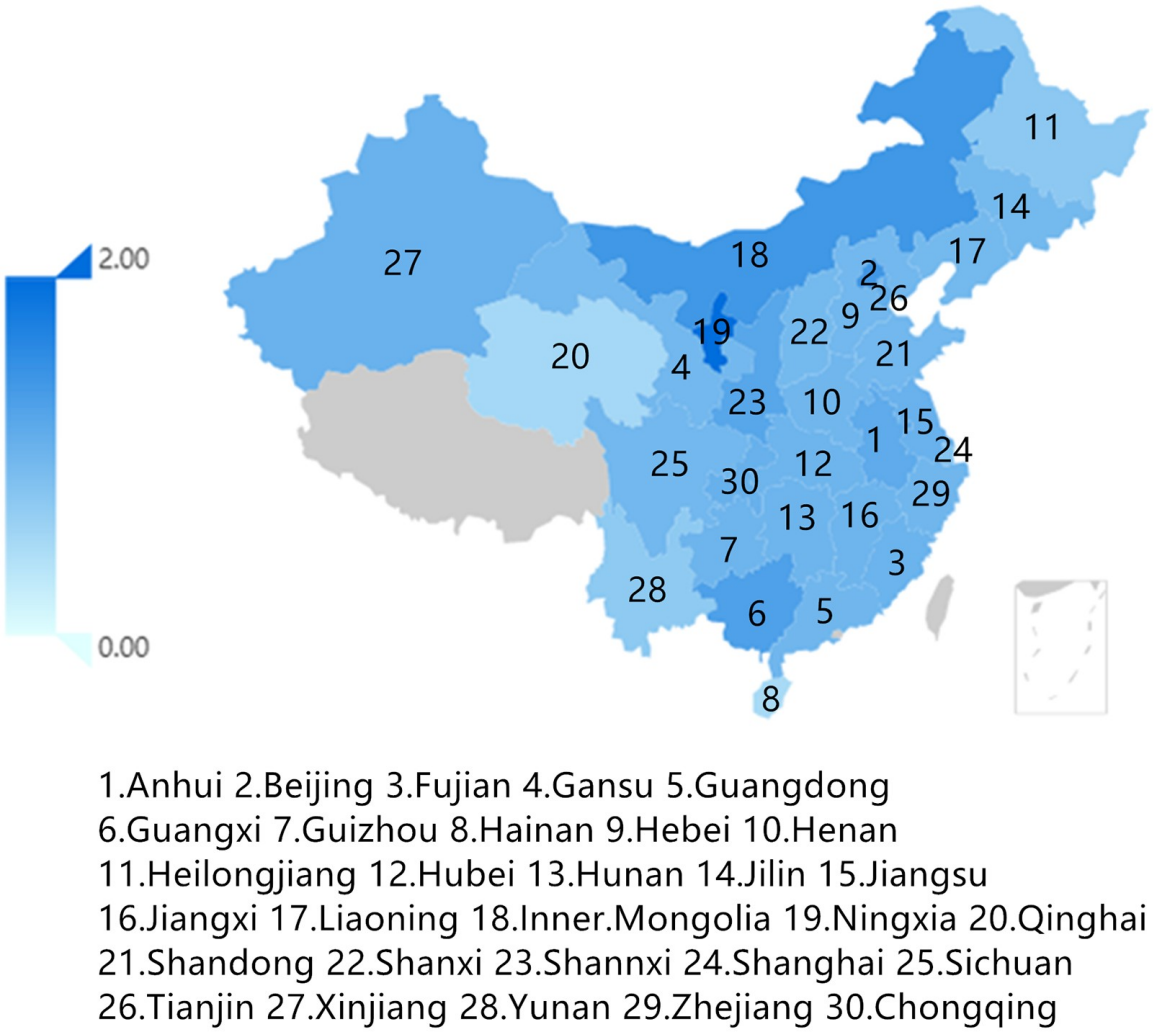
Change of output value of the equipment manufacturing industry in each province.

From the view of change in output value(Fig 4), the output values of industries in Ningxia, Inner Mongolia, Beijing, and other provinces and regions have increased apparently. However, the output values of industries in Yunnan, Shanghai, Qinghai, Hainan, and other provinces have decreased significantly. Overall, the output values of Central and Eastern provinces are relatively stable, and a substantial room for output improvement exists for Northern provinces.

The output values of Northeast and Northwest provinces have decreased markedly under the influence of the collaborative emissions reduction function, which is assumed to be related to the difference in production technology, energy structure, and pollution regulation intensity. Li (2017) declared that the existence of heterogeneity among provinces and regions cannot be ignored in the study of China’s carbon emissions, which requires further analysis of the industrial transfer trend of provincial industries and clarification of what is behind the direction adjustment of the spatial pattern of the provincial industry structure of the equipment manufacturing industry [33].

#### 4.4.3 Analysis of the change of subdividing industries in provinces and regions

Based on the analysis of the regional industries of the equipment manufacturing industry, a detailed comparative analysis is carried out on the industries in each province and region to reflect the regional heterogeneity characteristics brought about by the differences in the intensity of environmental control, energy endowment, and production technology of each province. At the same time, the current situation of industrial development in different regions can be analyzed from the perspective of the direction and quantity of industrial transfer, and specific guidance for industrial optimization, energy saving, and emissions reduction can be provided. Only typical provinces are analyzed, as the number of provinces and industries is large.

**Fig 5:**
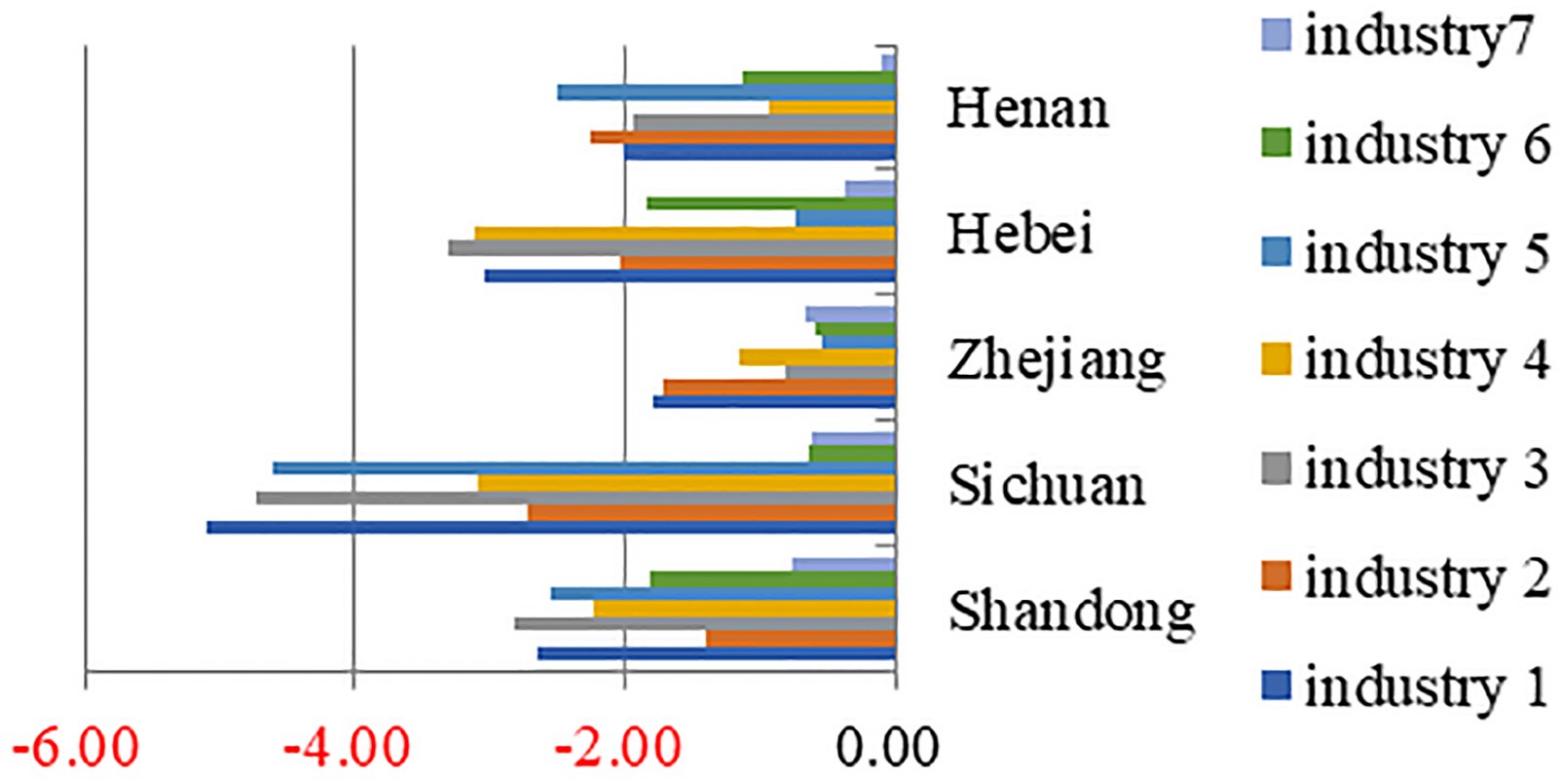
Typical provinces with output reduction.

**Fig 6:**
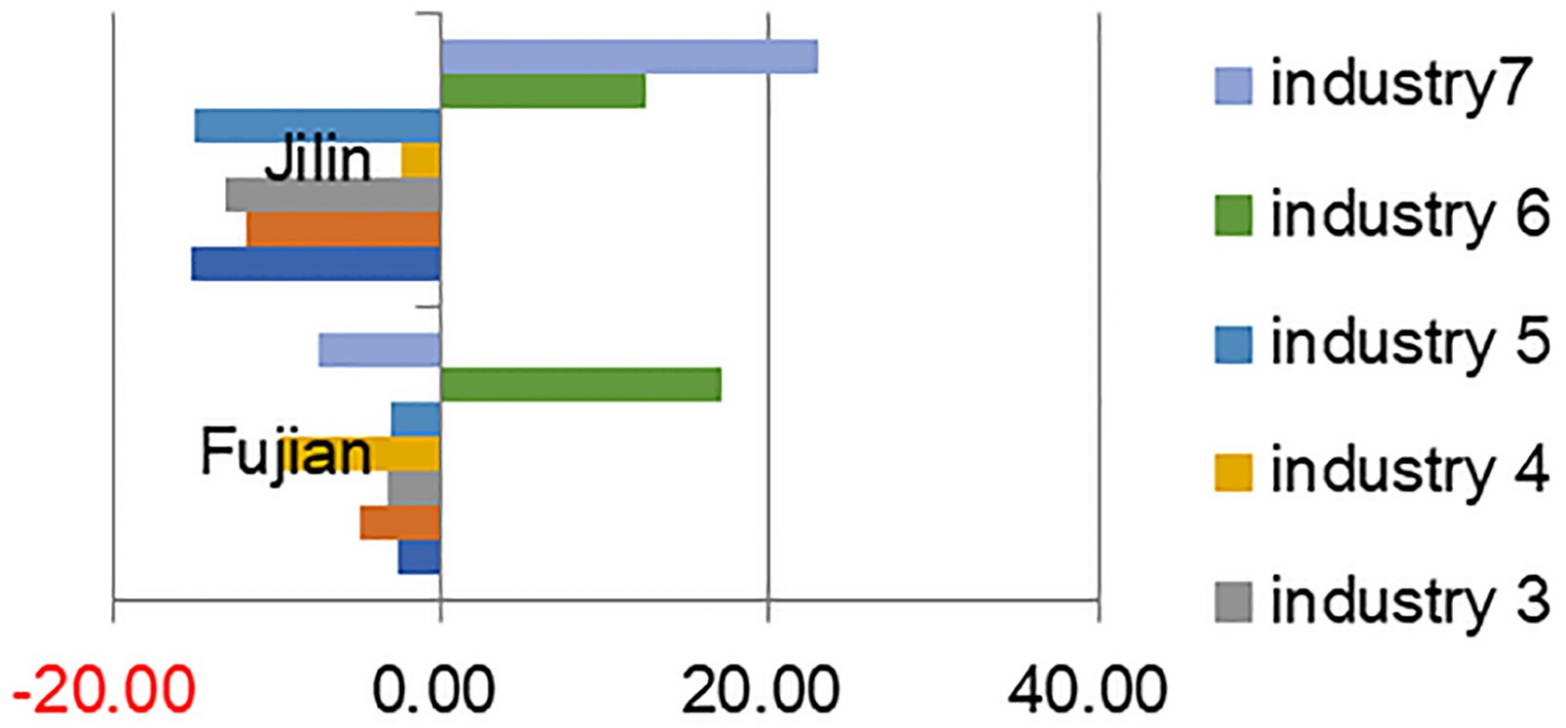
Typical provinces with increasing or decreasing industries.

**Fig 7:**
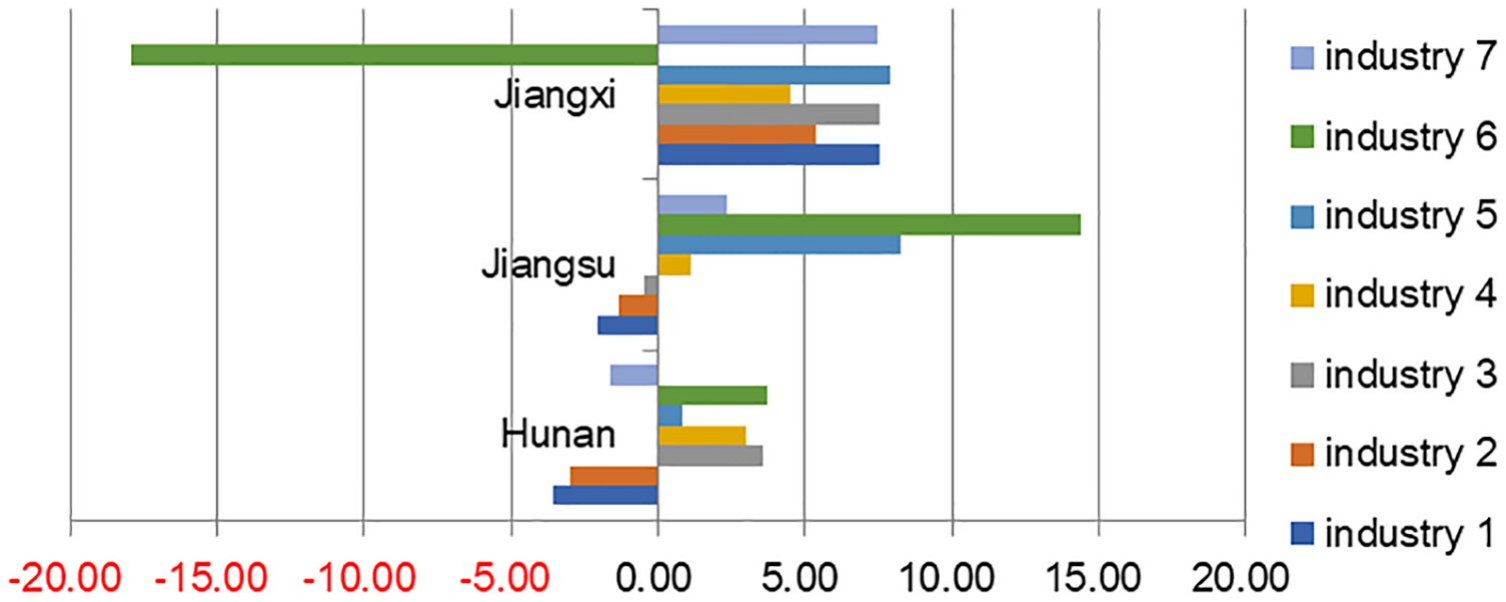
Typical provinces with output increase.

According to the optimization and adjustment results(Figs 5, 6 and 7), the output values of all the subdivided industries in the provinces of Zhejiang, Sichuan, Shandong, Henan, and Hebei have declined. In 12 provinces, such as Fujian and Jilin, only the output of one or two industries has increased, while the output values of the other industries have declined. However, the total output values of several provinces have decreased while those of a few have increased. For example, though only the output value of the electronics and telecommunications equipment industry has increased in Fujian Province, its total output value has increased significantly. At the same time, the output values of the electronic equipment industry and the instruments industry in Jilin province have increased to a certain extent, but the overall output value of the equipment manufacturing industry in Jilin Province has decreased.

#### 4.4.4 Discussion on industrial transfer under regional heterogeneity

Regional heterogeneity can be considered for industrial structure optimization to make the results specific to each subdivided industry of the provincial equipment manufacturing industry and to increase the practicality of the conclusions. In addition, the relative changes in the output scales of different provinces and regions represent the direction of industrial spatial transfer. According to theory of international trade, when different regions produce homogeneous products (assuming the absence of transportation costs), regional specialization will eventually be formed according to the comparative advantages of the different regions. This theory is verified in this study by the reduction of output value in most provinces and the growth of output value in a few provinces.

There were significant differences in energy structure and pollution emission intensity between provinces and regions in China [50]. Thus the direction of industrial transfer between provinces represents the difference in energy endowment and production technology between provinces and regions. The direction of industrial transfer and the specific provinces that industries transfer to are shown in Table 7. For example, the output values of Jiangxi, Jiangsu, and Hunan Provinces have increased and become the direction of industrial transfer. Given that industrial optimization aims at collaborative emissions reduction, the growth of output value shows that these provinces have comparative advantages in energy structure and emissions reduction technology in the corresponding industries. At the same time, the output scales of Hebei and Shandong Provinces in Table 7 have decreased because the industries have transferred to other provinces. This finding indicates that these two provinces have comparative disadvantages in energy structure and emissions reduction technology in corresponding the industries. DimitriosPappas et al. (2017) analyzed the manufacturing transfer from China to Southeast Asia [39]. It is found that due to the influence of production technology, energy structure and other factors, the carbon emission intensity of the manufacturing industry in Southeast Asia is about 2-3 times that of China, posing a threat to the future environment of Asia. In general, the results of this paper prove that the overall emission intensity of China’s equipment manufacturing industry can be effectively reduced by transferring the industry to a province with higher production technology and a more reasonable energy structure, which has similarities with it.

**Table 7:** Spatial transfer direction of the equipment manufacturing industry and specific provinces undertaking industrial transfer.

Industry	Direction of transfer
Metal Products	Gansu; Hainan; Jiangxi; Inner Mongolia; Shaanxi; Shanghai
Ordinary Machinery	Jiangxi; Inner Mongolia; Shaanxi; Yunnan
Equipment for Special Purposes	Hainan; Hunan; Jiangxi; Qinghai; Xinjiang
Transportation Equipment	Anhui; Beijing; Hunan; Jiangsu; Jiangxi; Ningxia; Tianjin; Chongqing
Electric Equipment and Machinery	Anhui; Beijing; Gansu; Guangxi; Guizhou; Hunan; Hubei; Jiangsu; Jiangxi; Ningxia; Shanxi; Shanghai; Xinjiang; Chongqing
Electronic and Telecommunications Equipment	Anhui; Beijing; Fujian; Gansu; Guangdong; Guangxi; Guizhou; Hainan; Heilongjiang; Hubei; Hunan; Jilin; Jiangsu; Qinghai; Tianjin; Xinjiang; Yunnan; Chongqing
Instruments, Meters, Cultural and Office Machinery	Gansu; Heilongjiang; Jilin; Jiangsu; Jiangxi; Liaoning; Ningxia; Shanxi; Shanghai; Tianjin; Yunnan

The first column of Table 7 lists the names of the industries, and the second column shows the transfer direction of the equipment manufacturing industry in each province. This table can provide a theoretical reference for industry development and industrial transfer in the equipment manufacturing industry and also promote rationalization of the spatial layout of the regional industries.

### 4.5 Capital matching under optimal industrial structure

#### 4.5.1 Estimation results of production function

The estimation method and function form are validated before the production function is estimated (Table 8). The results show that except for *LogL*^2^, the other variables are significant at the 5% level. The likelihood ratio test value is approximately 961, and the *γ* value is 0.74164, which suggests that significant technical inefficiency exists and proves the applicability and necessity of the stochastic frontier function.

**Table 8:** Function estimation results.

LogY	Coef.	St.Err.	t-value	p-value	[95% Conf	Interval]	Sig
LogK	0.068	0.023	2.94	0.003	0.023	0.113	***
LogL	0.078	0.008	9.58	0.000	0.062	0.094	***
LogM	0.909	0.021	43.42	0.000	0.868	0.950	***
T	0.031	0.009	3.59	0.000	0.014	0.048	***
T^2^	0.002	0.001	2.14	0.032	0.000	0.004	**
LogK•LogK	0.048	0.004	12.51	0.000	0.041	0.056	***
LogM•Log M	0.097	0.004	27.67	0.000	0.090	0.104	***
LogK•LogL	0.039	0.004	8.77	0.000	0.030	0.048	***
LogK•Log M	-0.144	0.007	-19.60	0.000	-0.159	-0.130	***
LogL•Log M	-0.050	0.004	-11.81	0.000	-0.058	-0.041	***
T•LogK	0.004	0.002	2.06	0.039	0.000	0.008	**
T•LogL	0.005	0.001	3.43	0.001	0.002	0.008	***
T•LogM	-0.004	0.002	2.35	0.019	-0.008	-0.001	**
Constant	0.528	0.057	9.27	0.000	0.416	0.639	***
Mean dependent var		5.168	SD dependent var		2.213		
Number of obs		2085.000	Chi-square		111086.049		
Prob > chi2		0.000	Akaike crit. (AIC)		-1886.075		

*** p<0.01,

** p<0.05,

* p<0.1

The reasonable allocation of capital factors is estimated according to optimized industrial output and input scale of production factors after the nonlinear relationship between production factors and the output scale is estimated. The original values of employment intensity and intermediate product input intensity in 2015 are used to calculate employment and intermediate product input to better solve the problem of capital mismatch between the industries and regions. On the one hand, the original value can ensure employment similar to the aforementioned nonlinear programming. On the other hand, the original value also corresponds to the characteristic of invariable equipment manufacturing technology, which makes capital matching reasonable after industrial structure optimization.

#### 4.5.2 Result and discussion of capital factor matching

Sustained capital investment is necessary to ensure technological innovation efficiency owing to the technology-intensive characteristic of the equipment manufacturing industry [52]. In addition to domestic investment, the equipment manufacturing industry also attracts considerable international investment. In recent years, foreign direct investment has accounted for more than 15%. Given the continuous growth of domestic and foreign investments, large capital redundancy and capital mismatch have formed in the Chinese equipment manufacturing industry [7].

**Fig 8:**
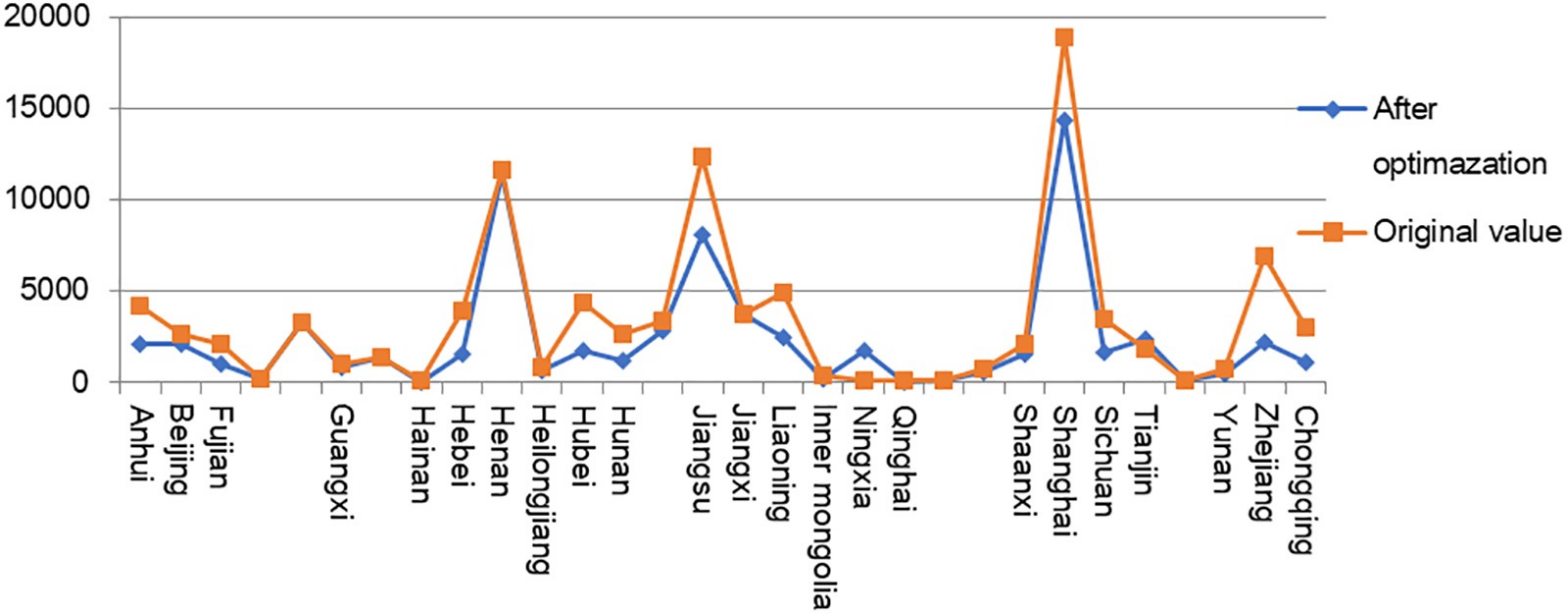
Capital changes in provinces and regions.

According to Table 9, reasonable capital stock in the Chinese equipment manufacturing industry as a whole should be 7.14 trillion yuan, which is approximately 29.37% lower than the original capital stock of 10.1 trillion yuan. Except for the instrument manufacturing industry, the other industries need to reduce capital investment to varying degrees, while the transportation equipment manufacturing industry, the ordinary machinery industry, and the metal products industry must reduce capital investment substantially (Table 9). Capital investment in these three industries is not fully exploited and thus has caused capital redundancy. From the provincial point of view, excess capital exists widely among provinces, especially in Jiangsu, Shanghai, Zhejiang and others where the output scale of the equipment manufacturing industry is large. The capital input levels of these provinces and regions show a significant decreasing trend after optimization, while the capital input amounts of Liaoning, Ningxia, and other Northeast and Western provinces with relatively small output scales of the equipment manufacturing industry show a significant increase (see Fig 8).

**Table 9:** Optimized results of industrial capital mismatch.

Industry	Original value	After optimization	Reduced proportions
Metal Products	0.90	0.57	0.3596
Ordinary Machinery	1.46	0.90	0.3808
Equipment for Special Purposes	1.15	0.89	0.2247
Transportation Equipment	2.88	1.62	0.4369
Electric Equipment and Machinery	1.59	1.18	0.2592
Electronic and Telecommunications Equipment	1.84	1.47	0.2017
Instruments, Meters, Cultural and Office Machinery	0.29	0.50	-0.7138
Gross value	10.11	7.14	0.2937

**Fig 9:**
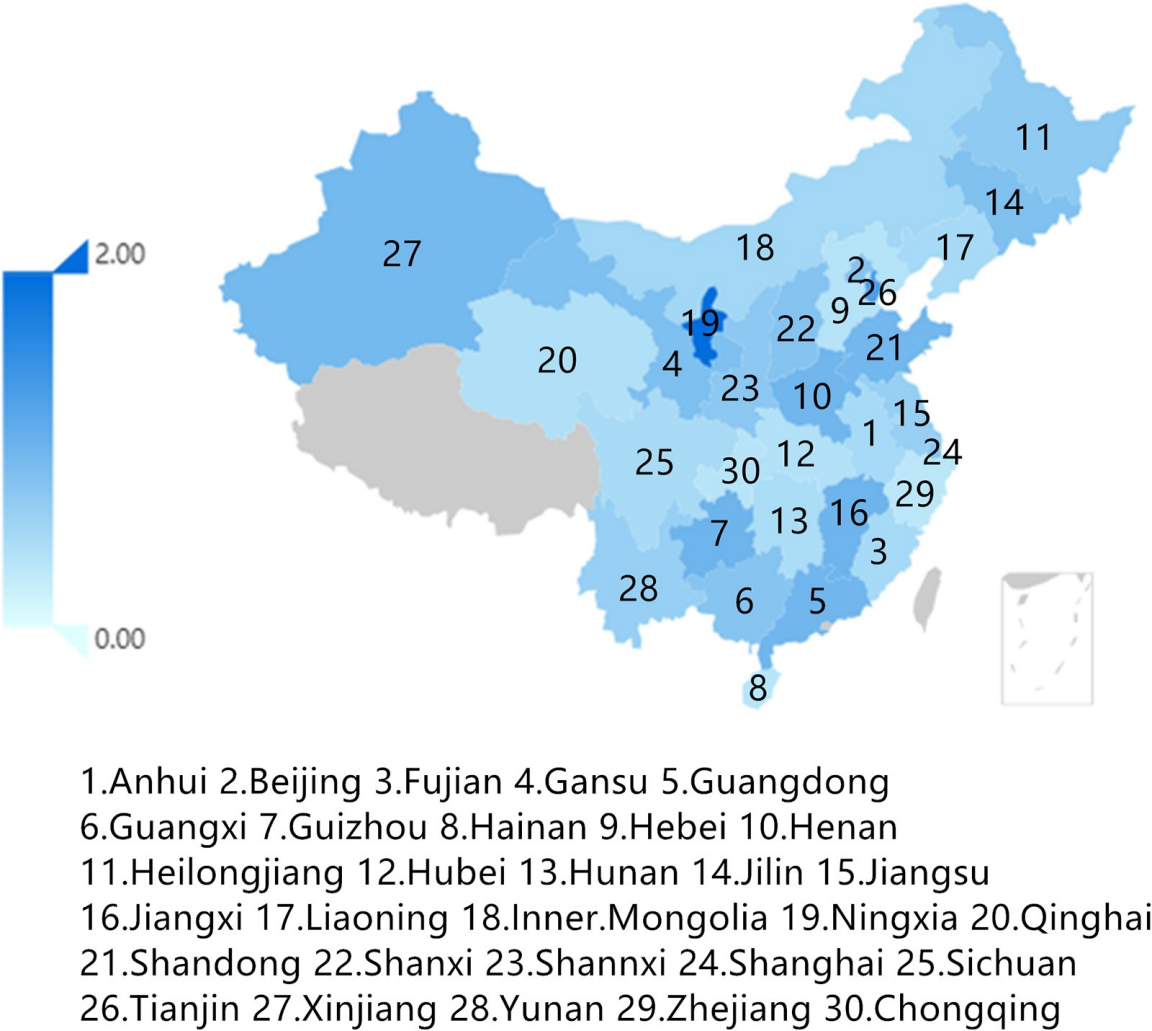
Regional capital allocation optimization.

Compared with the result of industrial transfer, that of capital flow shows a certain centralized trend in the entire country, that is, the reasonable capital stock in western and central north provinces and regions has increased comparatively, whereas the reasonable capital stock in most southern provinces and regions is significantly lower than the actual level. As shown in Fig 9, the capital investment of most provinces must be reduced while that of certain provinces should be increased. This study provides a reference for industrial capital transfer to each province and region by analyzing the trend of capital transfer from the perspective of heterogeneity to reduce redundant capital and excess capacity. This reduction can also help achieve high output efficiency and low pollution intensity. Generally, the conclusion could promote efficiency of capital allocation and further promote systematic optimization of industrial structure.

Analysis of the matching capital level could provide a theoretical basis for solving the industrial and regional mismatch of capital simultaneously as well as a reference for the reasonable capital stock levels of provincial and regional industries. There is a high correlation between the capital input and the output scale, and the reasonable industrial investment is the key to realizing industrial transfer [53]. In addition, the high capital investment in the manufacturing industry is one of the leading causes of environmental pollution [54]. Therefore, reasonable capital allocation is one of the key factors to realize the stereoscopic optimization of collaborative emission reduction, industrial structure optimization and production factor allocation. The research conclusion largely met the theoretical expectation.

## 5 Conclusions

This study employs a nonlinear programming method and capital matching using a panel data of 30 Chinese provinces from 2006 to 2015 to solve the problems of high pollution intensity, unreasonable industrial structure, and overcapacity in the equipment manufacturing industry. Compared with previous literature, the contribution of this paper is as follows: (1) on the basis of carbon emission reduction, this paper expands the collaborative emission reduction of *SO*_2_ and *CO*_2_, which provides a more satisfactory result, that is, it measures the pollution level and the potential of energy-saving and emission reduction of China’s equipment manufacturing industry more accurately.

Analysis of the equipment manufacturing industry, as a sample, has a certain reference value for manufacturing industries in other countries and regions. Empirical studies in China, Finland and other countries show that industry heterogeneity and regional heterogeneity exist extensively [55, 56], which play an important role in the evolution of industrial structure [44]. Meanwhile, capital input will also cast a long-term effect on the emission of manufacturing industries [27, 28, 53]. Therefore, the energy-saving and the emissions reduction effect of industrial structure adjustment should not be ignored and adjusting the corresponding level of capital allocation is necessary. However, to achieve the transfer of the equipment manufacturing industry, mentioned in the conclusion of this paper, might be confronted with challenges. The study of Wang et al. (2018) points out that industries with high pollution tend to move to areas with relatively lax environmental regulations, forming a pollution haven effect [57]. Therefore, in the process of industrial transfer, the provinces undertaking the industries should strengthen the evaluation of environmental benefits or costs and pay attention to solving the problem of employment in the transferred areas.

Accordingly, we put forward the following policy suggestions. Firstly, when formulating environmental policies, all provinces and regions should take the collaborative emission reduction effect into consideration and propose regulations or emission reduction policies that are applicable to a wide range of pollutants. Secondly, in order to realize the optimal inter-regional industrial allocation in the conclusion of this paper, aside from formulating relevant policies to guide the industrial transfer, the provinces and regions should first ensure that there are no significant differences in environmental regulation, so as to avoid the evasive transfer of heavy polluting enterprises. At the same time, the provinces and regions that undertake the transfer should strengthen the environmental supervision of related industries, and lay emphasis on dissolving the environmental externalities and social costs caused by the industrial transfer, so as to solve the employment. Thirdly, all provinces and regions should pay attention to avoiding excessive long-term investment in the industry. Relevant authorities should try to reduce the capital redundancy in the industry by raising the marginal cost of pollution, restricting pollution investment and other fiscal policies while ensuring production capacity and economic stability.

In general, this study also has several limitations. For example, other pollutants such as *NO*_*x*_,*CO* and *VOC* were not included in the analysis. Further research can take other pollutants into consideration by expanding emission equation or constructing other comprehensive pollution indicators. Besides, the method of using historical data to calculate matching capital allocation may only reduce capital stock to the historical average level; thus, the potential for capital adjustment exists.

## Supporting information

S1 File (ZIP)
