# Supplementary material for: Stereoscopic optimization of industrial structure of the equipment manufacturing industry from the perspective of collaborative emissions reduction: Evidence from China
Source: PLoS One. 2020 Apr 30;15(4):e0232293. doi: 10.1371/journal.pone.0232293 (PMC7192503; doi:10.1371/journal.pone.0232293)
Supplement: S1 File — (ZIP) [file pone.0232293.s001.zip › supporting information/S3 nonlinearcondition of matlab.docx]

ceq(1)=x(1)*548.9900677-x(210);

ceq(2)=x(2)*660.9748888-x(211);

ceq(3)=x(3)*412.8665681-x(212);

ceq(4)=x(4)*474.2681793-x(213);

ceq(5)=x(5)*353.4930124-x(214);

ceq(6)=x(6)*641.996508-x(215);

ceq(7)=x(7)*208.7754625-x(216);

ceq(8)=x(8)*1034.930299-x(217);

ceq(9)=x(9)*562.8925586-x(218);

ceq(10)=x(10)*386.9392491-x(219);

ceq(11)=x(11)*273.3730927-x(220);

ceq(12)=x(12)*241.5610056-x(221);

ceq(13)=x(13)*347.3444288-x(222);

ceq(14)=x(14)*299.8554605-x(223);

ceq(15)=x(15)*287.7980459-x(224);

ceq(16)=x(16)*199.3408457-x(225);

ceq(17)=x(17)*142.9829736-x(226);

ceq(18)=x(18)*201.4597933-x(227);

ceq(19)=x(19)*161.7471958-x(228);

ceq(20)=x(20)*121.7861994-x(229);

ceq(21)=x(21)*163.725327-x(230);

ceq(22)=x(22)*1079.080189-x(231);

ceq(23)=x(23)*1461.063117-x(232);

ceq(24)=x(24)*780.1293451-x(233);

ceq(25)=x(25)*486.1477466-x(234);

ceq(26)=x(26)*473.3652721-x(235);

ceq(27)=x(27)*835.1074901-x(236);

ceq(28)=x(28)*1000.307715-x(237);

ceq(29)=x(29)*1708.071559-x(238);

ceq(30)=x(30)*1211.761197-x(239);

ceq(31)=x(31)*1213.248465-x(240);

ceq(32)=x(32)*2140.698398-x(241);

ceq(33)=x(33)*785.739039-x(242);

ceq(34)=x(34)*930.1449839-x(243);

ceq(35)=x(35)*2351.318173-x(244);

ceq(36)=x(36)*1424.157077-x(245);

ceq(37)=x(37)*1001.844394-x(246);

ceq(38)=x(38)*434.3056996-x(247);

ceq(39)=x(39)*429.2203934-x(248);

ceq(40)=x(40)*568.1519547-x(249);

ceq(41)=x(41)*143.0281175-x(250);

ceq(42)=x(42)*438.5973335-x(251);

ceq(43)=x(43)*6425.229682-x(252);

ceq(44)=x(44)*2620.501666-x(253);

ceq(45)=x(45)*388.3572856-x(254);

ceq(46)=x(46)*634.6539739-x(255);

ceq(47)=x(47)*862.029129-x(256);

ceq(48)=x(48)*332.7705037-x(257);

ceq(49)=x(49)*935.6526933-x(258);

ceq(50)=x(50)*1019.379444-x(259);

ceq(51)=x(51)*3253.219864-x(260);

ceq(52)=x(52)*189.7517927-x(261);

ceq(53)=x(53)*299.4541818-x(262);

ceq(54)=x(54)*1213.422281-x(263);

ceq(55)=x(55)*255.2200503-x(264);

ceq(56)=x(56)*29.40008278-x(265);

ceq(57)=x(57)*673.7813844-x(266);

ceq(58)=x(58)*486.2979787-x(267);

ceq(59)=x(59)*471.5463828-x(268);

ceq(60)=x(60)*371.3962024-x(269);

ceq(61)=x(61)*327.1550713-x(270);

ceq(62)=x(62)*340.553675-x(271);

ceq(63)=x(63)*173.3333997-x(272);

ceq(64)=x(64)*770.3937219-x(273);

ceq(65)=x(65)*519.51388-x(274);

ceq(66)=x(66)*479.3879022-x(275);

ceq(67)=x(67)*678.7183057-x(276);

ceq(68)=x(68)*547.7877487-x(277);

ceq(69)=x(69)*265.0989341-x(278);

ceq(70)=x(70)*409.9127082-x(279);

ceq(71)=x(71)*1903.987681-x(280);

ceq(72)=x(72)*2813.527428-x(281);

ceq(73)=x(73)*778.0623505-x(282);

ceq(74)=x(74)*2268.308758-x(283);

ceq(75)=x(75)*1026.506654-x(284);

ceq(76)=x(76)*1012.149053-x(285);

ceq(77)=x(77)*505.5361327-x(286);

ceq(78)=x(78)*958.4950654-x(287);

ceq(79)=x(79)*775.6786351-x(288);

ceq(80)=x(80)*776.7519892-x(289);

ceq(81)=x(81)*816.2735343-x(290);

ceq(82)=x(82)*786.7470604-x(291);

ceq(83)=x(83)*330.3701783-x(292);

ceq(84)=x(84)*460.6930141-x(293);

ceq(85)=x(85)*2238.702831-x(294);

ceq(86)=x(86)*657.7268949-x(295);

ceq(87)=x(87)*384.9026961-x(296);

ceq(88)=x(88)*493.066962-x(297);

ceq(89)=x(89)*468.9641676-x(298);

ceq(90)=x(90)*754.8015507-x(299);

ceq(91)=x(91)*495.4429796-x(300);

ceq(92)=x(92)*954.2697356-x(301);

ceq(93)=x(93)*508.4369512-x(302);

ceq(94)=x(94)*616.760007-x(303);

ceq(95)=x(95)*583.1435414-x(304);

ceq(96)=x(96)*842.3668601-x(305);

ceq(97)=x(97)*4710.381155-x(306);

ceq(98)=x(98)*429.4639017-x(307);

ceq(99)=x(99)*1278.910867-x(308);

ceq(100)=x(100)*285.0354594-x(309);

ceq(101)=x(101)*122.6499285-x(310);

ceq(102)=x(102)*106.4958747-x(311);

ceq(103)=x(103)*68.25199023-x(312);

ceq(104)=x(104)*65.84336779-x(313);

ceq(105)=x(105)*57.94163445-x(314);

ceq(106)=x(106)*1019.073563-x(315);

ceq(107)=x(107)*415.8010961-x(316);

ceq(108)=x(108)*621.964204-x(317);

ceq(109)=x(109)*844.6292445-x(318);

ceq(110)=x(110)*639.5432626-x(319);

ceq(111)=x(111)*477.0164863-x(320);

ceq(112)=x(112)*508.9487451-x(321);

ceq(113)=x(113)*1069.100111-x(322);

ceq(114)=x(114)*1167.840285-x(323);

ceq(115)=x(115)*1285.317217-x(324);

ceq(116)=x(116)*484.3184992-x(325);

ceq(117)=x(117)*802.4957442-x(326);

ceq(118)=x(118)*592.164077-x(327);

ceq(119)=x(119)*556.7037367-x(328);

ceq(120)=x(120)*1229.062631-x(329);

ceq(121)=x(121)*435.9912206-x(320);

ceq(122)=x(122)*588.9173135-x(331);

ceq(123)=x(123)*415.340203-x(332);

ceq(124)=x(124)*543.446082-x(333);

ceq(125)=x(125)*3252.155731-x(334);

ceq(126)=x(126)*52.65316496-x(335);

ceq(127)=x(127)*1216.183962-x(336);

ceq(128)=x(128)*637.6052776-x(337);

ceq(129)=x(129)*1025.574635-x(338);

ceq(130)=x(130)*200.8796064-x(339);

ceq(131)=x(131)*358.440029-x(340);

ceq(132)=x(132)*167.2649589-x(341);

ceq(133)=x(133)*3506.182278-x(342);

ceq(134)=x(134)*1497.001468-x(343);

ceq(135)=x(135)*543.8396867-x(344);

ceq(136)=x(136)*1079.86247-x(345);

ceq(137)=x(137)*2008.717662-x(346);

ceq(138)=x(138)*700.3797289-x(347);

ceq(139)=x(139)*5987.709176-x(348);

ceq(140)=x(140)*429240.8811-x(349);

ceq(141)=x(141)*212675.9457-x(350);

ceq(142)=x(142)*497797.2212-x(351);

ceq(143)=x(143)*383067.7839-x(352);

ceq(144)=x(144)*75192.91587-x(353);

ceq(145)=x(145)*193215.9872-x(354);

ceq(146)=x(146)*58445.92893-x(355);

ceq(147)=x(147)*7596.337618-x(356);

ceq(148)=x(148)*1271.631931-x(357);

ceq(149)=x(149)*1170.630842-x(358);

ceq(150)=x(150)*1208.621816-x(359);

ceq(151)=x(151)*349.1116283-x(360);

ceq(152)=x(152)*997.3368493-x(361);

ceq(153)=x(153)*124.7383585-x(362);

ceq(154)=x(154)*138.797349-x(363);

ceq(155)=x(155)*63.85951673-x(364);

ceq(156)=x(156)*100.2340336-x(365);

ceq(157)=x(157)*110.1746516-x(366);

ceq(158)=x(158)*69.05193458-x(367);

ceq(159)=x(159)*73.66665233-x(368);

ceq(160)=x(160)*389.2704944-x(369);

ceq(161)=x(161)*4159.042154-x(370);

ceq(162)=x(162)*4159.042154-x(371);

ceq(163)=x(163)*4159.042154-x(372);

ceq(164)=x(164)*4159.042154-x(373);

ceq(165)=x(165)*4159.042154-x(374);

ceq(166)=x(166)*4159.042154-x(375);

ceq(167)=x(167)*4159.042154-x(376);

ceq(168)=x(168)*3923.104495-x(377);

ceq(169)=x(169)*3923.104495-x(378);

ceq(170)=x(170)*3923.104495-x(379);

ceq(171)=x(171)*3923.104495-x(380);

ceq(172)=x(172)*3923.104495-x(381);

ceq(173)=x(173)*3923.104495-x(382);

ceq(174)=x(174)*3923.104495-x(383);

ceq(175)=x(175)*478.6480496-x(384);

ceq(176)=x(176)*727.3072742-x(385);

ceq(177)=x(177)*990.6968816-x(386);

ceq(178)=x(178)*81.02495119-x(387);

ceq(179)=x(179)*562.8077368-x(388);

ceq(180)=x(180)*359.4198909-x(389);

ceq(181)=x(181)*896.5222486-x(390);

ceq(182)=x(182)*1214.380697-x(391);

ceq(183)=x(183)*874.0560468-x(392);

ceq(184)=x(184)*1155.119186-x(393);

ceq(185)=x(185)*2261.13368-x(394);

ceq(186)=x(186)*223.821907-x(395);

ceq(187)=x(187)*10235.04263-x(396);

ceq(188)=x(188)*924.4121648-x(397);

ceq(189)=x(189)*654.9187341-x(398);

ceq(190)=x(190)*1750.49212-x(399);

ceq(191)=x(191)*591.0703811-x(400);

ceq(192)=x(192)*1914.259673-x(401);

ceq(193)=x(193)*1259.778699-x(402);

ceq(194)=x(194)*299.9943371-x(403);

ceq(195)=x(195)*1820.403162-x(404);

ceq(196)=x(196)*3223.181572-x(405);

ceq(197)=x(197)*3211.495774-x(406);

ceq(198)=x(198)*3211.554127-x(407);

ceq(199)=x(199)*3211.476723-x(408);

ceq(200)=x(200)*3211.490679-x(409);

ceq(201)=x(201)*3211.529038-x(410);

ceq(202)=x(202)*3211.419321-x(411);

ceq(203)=x(203)*1127.059752-x(412);

ceq(204)=x(204)*613.1415992-x(413);

ceq(205)=x(205)*544.4509512-x(414);

ceq(206)=x(206)*566.7488689-x(415);

ceq(207)=x(207)*326.8868433-x(416);

ceq(208)=x(208)*269.4482437-x(417);

ceq(209)=x(209)*317.6006542-x(418);

ceq(210)=x(1)*1448.5134138744-x(419);

ceq(211)=x(2)*2650.3229444671-x(420);

ceq(212)=x(3)*978.3513767472-x(421);

ceq(213)=x(4)*1266.9264991023-x(422);

ceq(214)=x(5)*590.9422416473-x(423);

ceq(215)=x(6)*277.3019823234-x(424);

ceq(216)=x(7)*118.4305620483-x(425);

ceq(217)=x(8)*2640.2838265051-x(426);

ceq(218)=x(9)*1284.8176026956-x(427);

ceq(219)=x(10)*998.7987650159-x(428);

ceq(220)=x(11)*447.5119719007-x(429);

ceq(221)=x(12)*522.3786495071-x(430);

ceq(222)=x(13)*192.2829599513-x(431);

ceq(223)=x(14)*864.1718203904-x(432);

ceq(224)=x(15)*1696.8966886267-x(433);

ceq(225)=x(16)*1769.8136917729-x(434);

ceq(226)=x(17)*1342.1570088330-x(435);

ceq(227)=x(18)*1983.4396882890-x(436);

ceq(228)=x(19)*1391.2625705441-x(437);

ceq(229)=x(20)*451.9730986198-x(438);

ceq(230)=x(21)*2561.5713141317-x(439);

ceq(231)=x(22)*4609.0690462777-x(440);

ceq(232)=x(23)*4879.8402295036-x(441);

ceq(233)=x(24)*4219.6158667052-x(442);

ceq(234)=x(25)*5275.1941093960-x(443);

ceq(235)=x(26)*1546.0892796865-x(444);

ceq(236)=x(27)*657.2138379326-x(445);

ceq(237)=x(28)*1085.5191129861-x(446);

ceq(238)=x(29)*3800.8409503262-x(447);

ceq(239)=x(30)*7158.0844481171-x(448);

ceq(240)=x(31)*1831.6411322787-x(449);

ceq(241)=x(32)*4359.2550791706-x(450);

ceq(242)=x(33)*1582.8015297331-x(451);

ceq(243)=x(34)*1011.0020589164-x(452);

ceq(244)=x(35)*5388.0934253605-x(453);

ceq(245)=x(36)*5571.6154421931-x(454);

ceq(246)=x(37)*5188.7450398328-x(455);

ceq(247)=x(38)*1766.5469578394-x(456);

ceq(248)=x(39)*1844.7911607835-x(457);

ceq(249)=x(40)*590.6300724522-x(458);

ceq(250)=x(41)*82.8205392416-x(459);

ceq(251)=x(42)*259.5331174648-x(460);

ceq(252)=x(43)*29750.8510516376-x(461);

ceq(253)=x(44)*3013.1795695836-x(462);

ceq(254)=x(45)*1295.9401713667-x(463);

ceq(255)=x(46)*2265.3211030798-x(464);

ceq(256)=x(47)*243.7763644759-x(465);

ceq(257)=x(48)*81.6496118605-x(466);

ceq(258)=x(49)*768.6870833987-x(467);

ceq(259)=x(50)*16923.9278550239-x(468);

ceq(260)=x(51)*992654.5545014870-x(469);

ceq(261)=x(52)*3853.0385858394-x(470);

ceq(262)=x(53)*4635.0345520799-x(471);

ceq(263)=x(54)*479.9580361046-x(472);

ceq(264)=x(55)*2947.4191631314-x(473);

ceq(265)=x(56)*117600.3311314910-x(474);

ceq(266)=x(57)*2973.6014242768-x(475);

ceq(267)=x(58)*19908.5727830285-x(476);

ceq(268)=x(59)*3966.1448068953-x(477);

ceq(269)=x(60)*2689.6381007727-x(478);

ceq(270)=x(61)*1457.4579217431-x(479);

ceq(271)=x(62)*4420.4851269468-x(480);

ceq(272)=x(63)*3936.4111902297-x(481);

ceq(273)=x(64)*3895.5389665532-x(482);

ceq(274)=x(65)*3908.0506370231-x(483);

ceq(275)=x(66)*7602.3169556408-x(484);

ceq(276)=x(67)*1419.0081628833-x(485);

ceq(277)=x(68)*4369.0176120601-x(486);

ceq(278)=x(69)*1697.4773348726-x(487);

ceq(279)=x(70)*1539.3086703409-x(488);

ceq(280)=x(71)*8285.3723664248-x(489);

ceq(281)=x(72)*15624.5799410634-x(490);

ceq(282)=x(73)*8433.1224021581-x(491);

ceq(283)=x(74)*18356.1286578867-x(492);

ceq(284)=x(75)*6471.9529339215-x(493);

ceq(285)=x(76)*877.3531169240-x(494);

ceq(286)=x(77)*1692.5270693538-x(495);

ceq(287)=x(78)*3579.7792597275-x(496);

ceq(288)=x(79)*29576.3048626637-x(497);

ceq(289)=x(80)*17884.6447947511-x(498);

ceq(290)=x(81)*1272.5475115494-x(499);

ceq(291)=x(82)*909.2373715061-x(500);

ceq(292)=x(83)*96.6176845332-x(501);

ceq(293)=x(84)*1717.2385954506-x(502);

ceq(294)=x(85)*3173.3781277019-x(503);

ceq(295)=x(86)*2571.3470431269-x(504);

ceq(296)=x(87)*737.8747479441-x(505);

ceq(297)=x(88)*370.3886860134-x(506);

ceq(298)=x(89)*1246.6791639684-x(507);

ceq(299)=x(90)*812.7418934793-x(508);

ceq(300)=x(91)*3731.9688241821-x(509);

ceq(301)=x(92)*8088.7712400168-x(510);

ceq(302)=x(93)*7466.5479072145-x(511);

ceq(303)=x(94)*6875.6135544960-x(512);

ceq(304)=x(95)*3028.6462724803-x(513);

ceq(305)=x(96)*12072.6832467430-x(514);

ceq(306)=x(97)*4658.0056399415-x(515);

ceq(307)=x(98)*5959.8884365908-x(516);

ceq(308)=x(99)*1963.8849336329-x(517);

ceq(309)=x(100)*5885.1328646774-x(518);

ceq(310)=x(101)*1321.3664668452-x(519);

ceq(311)=x(102)*1089.4528567040-x(520);

ceq(312)=x(103)*556.1350858452-x(521);

ceq(313)=x(104)*408.5473679431-x(522);

ceq(314)=x(105)*387.7201126491-x(523);

ceq(315)=x(106)*1461.1527746165-x(524);

ceq(316)=x(107)*2852.0231598841-x(525);

ceq(317)=x(108)*3130.6012066094-x(526);

ceq(318)=x(109)*2254.3536717543-x(527);

ceq(319)=x(110)*966.3079856888-x(528);

ceq(320)=x(111)*376.4599363565-x(529);

ceq(321)=x(112)*740.0577434207-x(530);

ceq(322)=x(113)*4079.4203211194-x(531);

ceq(323)=x(114)*10080.4814928943-x(532);

ceq(324)=x(115)*27977.2877563517-x(533);

ceq(325)=x(116)*1687.9005378836-x(534);

ceq(326)=x(117)*5274.3129042508-x(535);

ceq(327)=x(118)*2611.2187207447-x(536);

ceq(328)=x(119)*1387.0488099985-x(537);

ceq(329)=x(120)*572.0787832064-x(538);

ceq(330)=x(121)*2083.2588532099-x(539);

ceq(331)=x(122)*2425.6716484844-x(540);

ceq(332)=x(123)*143.4865938630-x(541);

ceq(333)=x(124)*1013.1624364952-x(542);

ceq(334)=x(125)*4037.1713480104-x(543);

ceq(335)=x(126)*11155.8893263419-x(544);

ceq(336)=x(127)*5835.2525997908-x(545);

ceq(337)=x(128)*6005.8324978837-x(546);

ceq(338)=x(129)*7040.6046870437-x(547);

ceq(339)=x(130)*8604.3431397674-x(548);

ceq(340)=x(131)*3407.9044508605-x(549);

ceq(341)=x(342)*15857.1977120684-x(550);

ceq(342)=x(133)*41695.5705618540-x(551);

ceq(343)=x(134)*24192.6526056690-x(552);

ceq(344)=x(135)*7202.3290687713-x(553);

ceq(345)=x(136)*1351.9454649589-x(554);

ceq(346)=x(137)*31682.7889060890-x(555);

ceq(347)=x(138)*6546.4905248534-x(556);

ceq(348)=x(139)*11833.8370505492-x(557);

ceq(349)=x(140)*696173.3408341470-x(558);

ceq(350)=x(141)*2802356.0644795900-x(559);

ceq(351)=x(142)*3308861.9486829000-x(560);

ceq(352)=x(143)*2915657.7327131000-x(561);

ceq(353)=x(144)*313970.3552700430-x(562);

ceq(354)=x(145)*1045278.5257383100-x(563);

ceq(355)=x(146)*385495.3710034300-x(564);

ceq(356)=x(147)*19601.8188160349-x(565);

ceq(357)=x(148)*6971.0047951965-x(566);

ceq(358)=x(149)*35722.4679267027-x(567);

ceq(359)=x(150)*7356.2087240873-x(568);

ceq(360)=x(151)*362.4588794629-x(569);

ceq(361)=x(152)*1311.5862506948-x(570);

ceq(362)=x(153)*733.9335307614-x(571);

ceq(363)=x(154)*1624.6963707125-x(572);

ceq(364)=x(155)*13125.6328601853-x(573);

ceq(365)=x(156)*2315.9587254813-x(574);

ceq(366)=x(157)*1668.5432308876-x(575);

ceq(367)=x(158)*1758.8935251688-x(576);

ceq(368)=x(159)*4422.5489433615-x(577);

ceq(369)=x(160)*7996.3575724815-x(578);

ceq(370)=x(161)*8098.8247448375-x(579);

ceq(371)=x(162)*22973.1235407104-x(580);

ceq(372)=x(163)*2459.3606236638-x(581);

ceq(373)=x(164)*2276.8861193447-x(582);

ceq(374)=x(165)*1664.2513735850-x(583);

ceq(375)=x(166)*563.7669891918-x(584);

ceq(376)=x(167)*701.4534063081-x(585);

ceq(377)=x(168)*8619.0644966382-x(586);

ceq(378)=x(169)*15833.4487966432-x(587);

ceq(379)=x(170)*12079.8709214177-x(588);

ceq(380)=x(171)*6165.1760699952-x(589);

ceq(381)=x(172)*6656.2326595063-x(590);

ceq(382)=x(173)*765.3793067514-x(591);

ceq(383)=x(174)*5931.8719051904-x(592);

ceq(384)=x(175)*2416.5247230006-x(593);

ceq(385)=x(176)*1958.5596472910-x(594);

ceq(386)=x(177)*2945.7589139674-x(595);

ceq(387)=x(178)*825.8387915345-x(596);

ceq(388)=x(179)*1381.8745460976-x(597);

ceq(389)=x(180)*308.6040407978-x(598);

ceq(390)=x(181)*1095.2102659146-x(599);

ceq(391)=x(182)*9131.3593671254-x(600);

ceq(392)=x(183)*3121.7154505636-x(601);

ceq(393)=x(184)*2753.6741506427-x(602);

ceq(394)=x(185)*7195.5006140892-x(603);

ceq(395)=x(186)*1334.9135794841-x(604);

ceq(396)=x(187)*12784.3845351825-x(605);

ceq(397)=x(188)*252456.9622186810-x(606);

ceq(398)=x(189)*2562.1860061588-x(607);

ceq(399)=x(190)*9066.1357790690-x(608);

ceq(400)=x(191)*2404.1903766169-x(609);

ceq(401)=x(192)*8227.4965929716-x(610);

ceq(402)=x(193)*1309.6200371113-x(611);

ceq(403)=x(194)*173.7119469678-x(612);

ceq(404)=x(195)*1077.1951208418-x(613);

ceq(405)=x(196)*3593.4123291160-x(614);

ceq(406)=x(197)*3655.9672456501-x(615);

ceq(407)=x(198)*2094.1899335646-x(616);

ceq(408)=x(199)*2226.3259392719-x(617);

ceq(409)=x(200)*1191.1005568920-x(618);

ceq(410)=x(201)*931.6396691525-x(619);

ceq(411)=x(202)*1400.2474451571-x(620);

ceq(412)=x(203)*5740.4225252447-x(621);

ceq(413)=x(204)*2593.4050072169-x(622);

ceq(414)=x(205)*803.7643453907-x(623);

ceq(415)=x(206)*1924.0049974065-x(624);

ceq(416)=x(207)*982.5603313452-x(625);

ceq(417)=x(208)*132.8512304228-x(626);

ceq(418)=x(209)*5531.6142069993-x(627);

ceq(419)=x(1)*1011.121184-x(628);

ceq(420)=x(2)*596.2517964-x(629);

ceq(421)=x(3)*878.48179-x(630);

ceq(422)=x(4)*471.8427633-x(631);

ceq(423)=x(5)*274.3066524-x(632);

ceq(424)=x(6)*645.1945422-x(633);

ceq(425)=x(7)*6452.230388-x(634);

ceq(426)=x(8)*3794.106765-x(635);

ceq(427)=x(9)*2281.478695-x(636);

ceq(428)=x(10)*2047.388645-x(637);

ceq(429)=x(11)*260.8772897-x(638);

ceq(430)=x(12)*1416.047274-x(639);

ceq(431)=x(13)*527.5556737-x(640);

ceq(432)=x(14)*4319.951549-x(641);

ceq(433)=x(15)*2149.707395-x(642);

ceq(434)=x(16)*2286.440238-x(643);

ceq(435)=x(17)*2865.671456-x(644);

ceq(436)=x(18)*1523.428569-x(645);

ceq(437)=x(19)*1315.068252-x(646);

ceq(438)=x(20)*695.068481-x(647);

ceq(439)=x(21)*11786.48163-x(648);

ceq(440)=x(22)*2779.236177-x(649);

ceq(441)=x(23)*4923.6583-x(650);

ceq(442)=x(24)*2737.754411-x(651);

ceq(443)=x(25)*8745.748856-x(652);

ceq(444)=x(26)*2535.529472-x(653);

ceq(445)=x(27)*5612.552603-x(654);

ceq(446)=x(28)*65983.26076-x(655);

ceq(447)=x(29)*2886.189916-x(656);

ceq(448)=x(30)*6515.12579-x(657);

ceq(449)=x(31)*6700.494717-x(658);

ceq(450)=x(32)*7212.441309-x(659);

ceq(451)=x(33)*1217.89489-x(660);

ceq(452)=x(34)*693.1222128-x(661);

ceq(453)=x(35)*32928.85997-x(662);

ceq(454)=x(36)*2130.104876-x(663);

ceq(455)=x(37)*2662.85835-x(664);

ceq(456)=x(38)*1719.228333-x(665);

ceq(457)=x(39)*343.4262983-x(666);

ceq(458)=x(40)*999.0241233-x(667);

ceq(459)=x(41)*698.1118311-x(668);

ceq(460)=x(42)*17675.27219-x(669);

ceq(461)=x(43)*4189.887883-x(670);

ceq(462)=x(44)*6822.353904-x(671);

ceq(463)=x(45)*6945.840848-x(672);

ceq(464)=x(46)*1877.151755-x(673);

ceq(465)=x(47)*3758.945064-x(674);

ceq(466)=x(48)*2553.561866-x(675);

ceq(467)=x(49)*55396.36628-x(676);

ceq(468)=x(50)*25271.00798-x(677);

ceq(469)=x(51)*1556686.83-x(678);

ceq(470)=x(52)*244241.2158-x(679);

ceq(471)=x(53)*7810.557881-x(680);

ceq(472)=x(54)*8920.077599-x(681);

ceq(473)=x(55)*44805.63551-x(682);

ceq(474)=x(56)*346638.736-x(683);

ceq(475)=x(57)*3169.270727-x(684);

ceq(476)=x(58)*6379.364885-x(685);

ceq(477)=x(59)*5864.286285-x(686);

ceq(478)=x(60)*3220.004325-x(687);

ceq(479)=x(61)*4275.367209-x(688);

ceq(480)=x(62)*16906.61987-x(689);

ceq(481)=x(63)*83166.13984-x(690);

ceq(482)=x(64)*7769.753322-x(691);

ceq(483)=x(65)*4661.788737-x(692);

ceq(484)=x(66)*4152.030323-x(693);

ceq(485)=x(67)*4060.995704-x(694);

ceq(486)=x(68)*4880.618121-x(695);

ceq(487)=x(69)*4086.791199-x(696);

ceq(488)=x(70)*37869.29625-x(697);

ceq(489)=x(71)*3668.09897-x(698);

ceq(490)=x(72)*1834.231773-x(699);

ceq(491)=x(73)*2155.500919-x(700);

ceq(492)=x(74)*1596.107493-x(701);

ceq(493)=x(75)*3050.777311-x(702);

ceq(494)=x(76)*26761.88068-x(703);

ceq(495)=x(77)*21819.49797-x(704);

ceq(496)=x(78)*2772.659913-x(705);

ceq(497)=x(79)*3054.572436-x(706);

ceq(498)=x(80)*3616.776352-x(707);

ceq(499)=x(81)*593.5388563-x(708);

ceq(500)=x(82)*2170.444382-x(709);

ceq(501)=x(83)*1881.086457-x(710);

ceq(502)=x(84)*20285.75214-x(711);

ceq(503)=x(85)*5344.469404-x(712);

ceq(504)=x(86)*3799.912737-x(713);

ceq(505)=x(87)*2260.353383-x(714);

ceq(506)=x(88)*2605.087175-x(715);

ceq(507)=x(89)*3454.618387-x(716);

ceq(508)=x(90)*3171.565696-x(717);

ceq(509)=x(91)*26096.6704-x(718);

ceq(510)=x(92)*2474.397451-x(719);

ceq(511)=x(93)*1825.109208-x(720);

ceq(512)=x(94)*1333.705813-x(721);

ceq(513)=x(95)*152.302672-x(722);

ceq(514)=x(96)*2273.122764-x(723);

ceq(515)=x(97)*9792.76365-x(724);

ceq(516)=x(98)*16357.83424-x(725);

ceq(517)=x(99)*995.8224109-x(726);

ceq(518)=x(100)*700.3195637-x(727);

ceq(519)=x(101)*1043.145311-x(728);

ceq(520)=x(102)*567.5023217-x(729);

ceq(521)=x(103)*379.7568071-x(730);

ceq(522)=x(104)*326.8914975-x(731);

ceq(523)=x(105)*1804.181184-x(732);

ceq(524)=x(106)*10465.24363-x(733);

ceq(525)=x(107)*9233.111578-x(734);

ceq(526)=x(108)*14296.52829-x(735);

ceq(527)=x(109)*5560.872992-x(736);

ceq(528)=x(110)*3167.168103-x(737);

ceq(529)=x(111)*4769.443568-x(738);

ceq(530)=x(112)*45890.98298-x(739);

ceq(531)=x(113)*1464.736683-x(740);

ceq(532)=x(114)*714.3519843-x(741);

ceq(533)=x(115)*1092.495159-x(742);

ceq(534)=x(116)*441.7907949-x(743);

ceq(535)=x(117)*1276.84147-x(744);

ceq(536)=x(118)*2908.932208-x(745);

ceq(537)=x(119)*10129.43965-x(746);

ceq(538)=x(120)*1884.352499-x(747);

ceq(539)=x(121)*2001.309138-x(748);

ceq(540)=x(122)*2384.823008-x(749);

ceq(541)=x(123)*2732.215425-x(750);

ceq(542)=x(124)*1514.985188-x(751);

ceq(543)=x(125)*8292.525525-x(752);

ceq(544)=x(126)*60181.11959-x(753);

ceq(545)=x(127)*8324.880362-x(754);

ceq(546)=x(128)*5524.894618-x(755);

ceq(547)=x(129)*8683.725433-x(756);

ceq(548)=x(130)*111467.424-x(757);

ceq(549)=x(131)*5109.754074-x(758);

ceq(550)=x(552)*33266.94198-x(759);

ceq(551)=x(133)*12042.49528-x(760);

ceq(552)=x(134)*10492.81595-x(761);

ceq(553)=x(135)*44748.31169-x(762);

ceq(554)=x(136)*20035.57771-x(763);

ceq(555)=x(137)*2673.331989-x(764);

ceq(556)=x(138)*38984.16568-x(765);

ceq(557)=x(139)*55821.08245-x(766);

ceq(558)=x(140)*952703.6988-x(767);

ceq(559)=x(141)*830104.9055-x(768);

ceq(560)=x(142)*3540089.394-x(769);

ceq(561)=x(143)*1585056.964-x(770);

ceq(562)=x(144)*211492.4356-x(771);

ceq(563)=x(145)*3084135.327-x(772);

ceq(564)=x(146)*4416161.684-x(773);

ceq(565)=x(147)*13529.81939-x(774);

ceq(566)=x(148)*7692.023303-x(775);

ceq(567)=x(149)*3067.067402-x(776);

ceq(568)=x(150)*6175.359494-x(777);

ceq(569)=x(151)*6484.603835-x(778);

ceq(570)=x(152)*6453.130268-x(779);

ceq(571)=x(153)*56671.67043-x(780);

ceq(572)=x(154)*7686.656988-x(781);

ceq(573)=x(155)*3579.982822-x(582);

ceq(574)=x(156)*2968.929222-x(783);

ceq(575)=x(157)*1399.797142-x(784);

ceq(576)=x(158)*2194.723358-x(785);

ceq(577)=x(159)*3626.946733-x(786);

ceq(578)=x(160)*24276.81934-x(787);

ceq(579)=x(161)*3672.894397-x(788);

ceq(580)=x(162)*1309.033793-x(789);

ceq(581)=x(163)*3160.267958-x(790);

ceq(582)=x(164)*545.5072036-x(791);

ceq(583)=x(165)*1530.691428-x(792);

ceq(584)=x(166)*618.4540961-x(793);

ceq(585)=x(167)*9917.080399-x(794);

ceq(586)=x(168)*6282.214253-x(795);

ceq(587)=x(169)*3401.517161-x(796);

ceq(588)=x(170)*5316.027607-x(797);

ceq(589)=x(171)*2216.109553-x(798);

ceq(590)=x(172)*5048.49109-x(799);

ceq(591)=x(173)*1631.673256-x(800);

ceq(592)=x(174)*84936.19981-x(801);

ceq(593)=x(175)*1454.165299-x(802);

ceq(594)=x(176)*1612.799751-x(803);

ceq(595)=x(177)*1582.260345-x(804);

ceq(596)=x(178)*539.4506404-x(805);

ceq(597)=x(179)*1687.872249-x(806);

ceq(598)=x(180)*742.3698354-x(807);

ceq(599)=x(181)*24725.26901-x(808);

ceq(600)=x(182)*5459.134818-x(809);

ceq(601)=x(183)*21146.93628-x(810);

ceq(602)=x(184)*10292.496-x(811);

ceq(603)=x(185)*22190.57485-x(812);

ceq(604)=x(186)*843.920016-x(813);

ceq(605)=x(187)*218762.4197-x(814);

ceq(606)=x(188)*357844.0575-x(815);

ceq(607)=x(189)*4911.119543-x(816);

ceq(608)=x(190)*9548.448449-x(817);

ceq(609)=x(191)*4640.614028-x(818);

ceq(610)=x(192)*2119.415858-x(819);

ceq(611)=x(193)*4334.639477-x(820);

ceq(612)=x(194)*17298.23568-x(821);

ceq(613)=x(195)*11953.09674-x(822);

ceq(614)=x(196)*5811.73795-x(823);

ceq(615)=x(197)*3358.416898-x(824);

ceq(616)=x(198)*8725.614886-x(825);

ceq(617)=x(199)*2848.713816-x(826);

ceq(618)=x(200)*2285.70628-x(827);

ceq(619)=x(201)*4974.019538-x(828);

ceq(620)=x(202)*17148.26569-x(829);

ceq(621)=x(203)*4189.805795-x(830);

ceq(622)=x(204)*3585.325256-x(831);

ceq(623)=x(205)*6076.483155-x(832);

ceq(624)=x(206)*407.7403438-x(833);

ceq(625)=x(207)*2213.727945-x(834);

ceq(626)=x(208)*743.7212195-x(835);

ceq(627)=x(209)*14939.55305-x(836);

c(1)=25988.04484-(x(1)+x(8)+x(15)+x(22)+x(29)+x(36)+x(43)+x(50)+x(57)+x(64)+x(71)+x(78)+x(85)+x(92)+x(99)+x(106)+x(113)+x(120)+x(127)+x(133)+x(140)+x(147)+x(154)+x(161)+x(168)+x(175)+x(182)+x(189)+x(196)+x(203));

c(2)=-32201.031997+(x(1)+x(8)+x(15)+x(22)+x(29)+x(36)+x(43)+x(50)+x(57)+x(64)+x(71)+x(78)+x(85)+x(92)+x(99)+x(106)+x(113)+x(120)+x(127)+x(133)+x(140)+x(147)+x(154)+x(161)+x(168)+x(175)+x(182)+x(189)+x(196)+x(203));

c(3)=24659.15341-(x(2)+x(9)+x(16)+x(23)+x(30)+x(37)+x(44)+x(51)+x(58)+x(65)+x(72)+x(79)+x(86)+x(93)+x(100)+x(107)+x(114)+x(121)+x(128)+x(134)+x(141)+x(148)+x(155)+x(162)+x(169)+x(176)+x(183)+x(190)+x(197)+x(204));

c(4)=-47682.81144+(x(2)+x(9)+x(16)+x(23)+x(30)+x(37)+x(44)+x(51)+x(58)+x(65)+x(72)+x(79)+x(86)+x(93)+x(100)+x(107)+x(114)+x(121)+x(128)+x(134)+x(141)+x(148)+x(155)+x(162)+x(169)+x(176)+x(183)+x(190)+x(197)+x(204));

c(5)=10256.2101-(x(3)+x(10)+x(17)+x(24)+x(31)+x(38)+x(45)+x(52)+x(59)+x(66)+x(73)+x(80)+x(87)+x(94)+x(101)+x(108)+x(115)+x(122)+x(129)+x(135)+x(142)+x(149)+x(156)+x(163)+x(170)+x(177)+x(184)+x(191)+x(198)+x(205));

c(6)=-37608.82327+(x(3)+x(10)+x(17)+x(24)+x(31)+x(38)+x(45)+x(52)+x(59)+x(66)+x(73)+x(80)+x(87)+x(94)+x(101)+x(108)+x(115)+x(122)+x(129)+x(135)+x(142)+x(149)+x(156)+x(163)+x(170)+x(177)+x(184)+x(191)+x(198)+x(205));

c(7)=42768.11747-(x(4)+x(11)+x(18)+x(25)+x(32)+x(39)+x(46)+x(53)+x(60)+x(67)+x(74)+x(81)+x(88)+x(95)+x(102)+x(109)+x(116)+x(123)+x(130)+x(136)+x(143)+x(150)+x(157)+x(164)+x(171)+x(178)+x(185)+x(192)+x(199)+x(206));

c(8)=-92872.05144+(x(4)+x(11)+x(18)+x(25)+x(32)+x(39)+x(46)+x(53)+x(60)+x(67)+x(74)+x(81)+x(88)+x(95)+x(102)+x(109)+x(116)+x(123)+x(130)+x(136)+x(143)+x(150)+x(157)+x(164)+x(171)+x(178)+x(185)+x(192)+x(199)+x(206));

c(9)=47713.41077-(x(5)+x(12)+x(19)+x(26)+x(33)+x(40)+x(47)+x(54)+x(61)+x(68)+x(75)+x(82)+x(89)+x(96)+x(103)+x(110)+x(117)+x(124)+x(131)+x(137)+x(144)+x(151)+x(158)+x(165)+x(172)+x(179)+x(186)+x(193)+x(200)+x(207));

c(10)=-71064.75007+(x(5)+x(12)+x(19)+x(26)+x(33)+x(40)+x(47)+x(54)+x(61)+x(68)+x(75)+x(82)+x(89)+x(96)+x(103)+x(110)+x(117)+x(124)+x(131)+x(137)+x(144)+x(151)+x(158)+x(165)+x(172)+x(179)+x(186)+x(193)+x(200)+x(207));

c(11)=65679.61478-(x(6)+x(13)+x(20)+x(27)+x(34)+x(41)+x(48)+x(55)+x(62)+x(69)+x(76)+x(83)+x(90)+x(97)+x(104)+x(111)+x(118)+x(125)+x(138)+x(145)+x(152)+x(159)+x(166)+x(173)+x(180)+x(187)+x(194)+x(201)+x(208));

c(12)=-127407.1307+(x(6)+x(13)+x(20)+x(27)+x(34)+x(41)+x(48)+x(55)+x(62)+x(69)+x(76)+x(83)+x(90)+x(97)+x(104)+x(111)+x(118)+x(125)+x(138)+x(145)+x(152)+x(159)+x(166)+x(173)+x(180)+x(187)+x(194)+x(201)+x(208));

c(13)=6069.649261-(x(7)+x(14)+x(21)+x(28)+x(35)+x(42)+x(49)+x(56)+x(63)+x(70)+x(77)+x(84)+x(91)+x(98)+x(105)+x(112)+x(119)+x(126)+x(132)+x(139)+x(146)+x(153)+x(160)+x(167)+x(174)+x(181)+x(188)+x(195)+x(202)+x(209));

c(14)=-15535.20568+(x(7)+x(14)+x(21)+x(28)+x(35)+x(42)+x(49)+x(56)+x(63)+x(70)+x(77)+x(84)+x(91)+x(98)+x(105)+x(112)+x(119)+x(126)+x(132)+x(139)+x(146)+x(153)+x(160)+x(167)+x(174)+x(181)+x(188)+x(195)+x(202)+x(209));

c(15)=-349094753.0051+(x(210)+x(211)+x(212)+x(213)+x(214)+x(215)+x(216)+x(217)+x(218)+x(219)+x(220)+x(221)+x(222)+x(223)+x(224)+x(225)+x(226)+x(227)+x(228)+x(229)+x(230)+x(231)+x(232)+x(233)+x(234)+x(235)+x(236)+x(237)+x(238)+x(239)+x(240)+x(241)+x(242)+x(243)+x(244)+x(245)+x(246)+x(247)+x(248)+x(249)+x(250)+x(251)+x(252)+x(253)+x(254)+x(255)+x(256)+x(257)+x(258)+x(259)+x(260)+x(261)+x(262)+x(263)+x(264)+x(265)+x(266)+x(267)+x(268)+x(269)+x(270)+x(271)+x(272)+x(273)+x(274)+x(275)+x(276)+x(277)+x(278)+x(279)+x(280)+x(281)+x(282)+x(283)+x(284)+x(285)+x(286)+x(287)+x(288)+x(289)+x(290)+x(291)+x(292)+x(293)+x(294)+x(295)+x(296)+x(297)+x(298)+x(299)+x(300)+x(301)+x(302)+x(303)+x(304)+x(305)+x(306)+x(307)+x(308)+x(309)+x(310)+x(311)+x(312)+x(313)+x(314)+x(315)+x(316)+x(317)+x(318)+x(319)+x(320)+x(321)+x(322)+x(323)+x(324)+x(325)+x(326)+x(327)+x(328)+x(329)+x(330)+x(331)+x(332)+x(333)+x(334)+x(335)+x(336)+x(337)+x(338)+x(339)+x(340)+x(341)+x(342)+x(343)+x(344)+x(345)+x(346)+x(347)+x(348)+x(349)+x(350)+x(351)+x(352)+x(353)+x(354)+x(355)+x(356)+x(357)+x(358)+x(359)+x(360)+x(361)+x(362)+x(363)+x(364)+x(365)+x(366)+x(367)+x(368)+x(369)+x(370)+x(371)+x(372)+x(373)+x(374)+x(375)+x(376)+x(377)+x(378)+x(379)+x(380)+x(381)+x(382)+x(383)+x(384)+x(385)+x(386)+x(387)+x(388)+x(389)+x(390)+x(391)+x(392)+x(393)+x(394)+x(395)+x(396)+x(397)+x(398)+x(399)+x(400)+x(401)+x(402)+x(403)+x(404)+x(405)+x(406)+x(407)+x(408)+x(409)+x(410)+x(411)+x(412)+x(413)+x(414)+x(415)+x(416)+x(417)+x(418));

c(16)=-909310440+(x(419)+x(420)+x(421)+x(422)+x(423)+x(424)+x(425)+x(426)+x(427)+x(428)+x(429)+x(430)+x(431)+x(432)+x(433)+x(434)+x(435)+x(436)+x(437)+x(438)+x(439)+x(440)+x(441)+x(442)+x(443)+x(444)+x(445)+x(446)+x(447)+x(448)+x(449)+x(450)+x(451)+x(452)+x(453)+x(454)+x(455)+x(456)+x(457)+x(458)+x(459)+x(460)+x(461)+x(462)+x(463)+x(464)+x(465)+x(466)+x(467)+x(468)+x(469)+x(470)+x(471)+x(472)+x(473)+x(474)+x(475)+x(476)+x(477)+x(478)+x(479)+x(480)+x(481)+x(482)+x(483)+x(484)+x(485)+x(486)+x(487)+x(488)+x(489)+x(490)+x(491)+x(492)+x(493)+x(494)+x(495)+x(496)+x(497)+x(498)+x(499)+x(500)+x(501)+x(502)+x(503)+x(504)+x(505)+x(506)+x(507)+x(508)+x(509)+x(510)+x(511)+x(512)+x(513)+x(514)+x(515)+x(516)+x(517)+x(518)+x(519)+x(520)+x(521)+x(522)+x(523)+x(524)+x(525)+x(526)+x(527)+x(528)+x(529)+x(530)+x(531)+x(532)+x(533)+x(534)+x(535)+x(536)+x(537)+x(538)+x(539)+x(540)+x(541)+x(542)+x(543)+x(544)+x(545)+x(546)+x(547)+x(548)+x(549)+x(550)+x(551)+x(552)+x(553)+x(554)+x(555)+x(556)+x(557)+x(558)+x(559)+x(560)+x(561)+x(562)+x(563)+x(564)+x(565)+x(566)+x(567)+x(568)+x(569)+x(570)+x(571)+x(572)+x(573)+x(574)+x(575)+x(576)+x(577)+x(578)+x(579)+x(580)+x(581)+x(582)+x(583)+x(584)+x(585)+x(586)+x(587)+x(588)+x(589)+x(590)+x(591)+x(592)+x(593)+x(594)+x(595)+x(596)+x(597)+x(598)+x(599)+x(600)+x(601)+x(602)+x(603)+x(604)+x(605)+x(606)+x(607)+x(608)+x(609)+x(610)+x(611)+x(612)+x(613)+x(614)+x(615)+x(616)+x(617)+x(618)+x(619)+x(620)+x(621)+x(622)+x(623)+x(624)+x(625)+x(626)+x(627));

c(17)=8195443-(x(628)+x(629)+x(630)+x(631)+x(632)+x(633)+x(634));

c(18)=-8737291+(x(628)+x(629)+x(630)+x(631)+x(632)+x(633)+x(634));

c(19)=8063440-(x(635)+x(636)+x(637)+x(638)+x(639)+x(640)+x(641));

c(20)=-8596560+(x(635)+x(636)+x(637)+x(638)+x(639)+x(640)+x(641));

c(21)=15955447-(x(642)+x(643)+x(644)+x(645)+x(646)+x(647)+x(648));

c(22)=-17010353+(x(642)+x(643)+x(644)+x(645)+x(646)+x(647)+x(648));

c(23)=2413611-(x(649)+x(650)+x(651)+x(652)+x(653)+x(654)+x(655));

c(24)=-2573189+(x(649)+x(650)+x(651)+x(652)+x(653)+x(654)+x(655));

c(25)=66473238-(x(656)+x(657)+x(658)+x(659)+x(660)+x(661)+x(662));

c(26)=-70868162+(x(656)+x(657)+x(658)+x(659)+x(660)+x(661)+x(662));

c(27)=5163888-(x(663)+x(664)+x(665)+x(666)+x(667)+x(668)+x(669));

c(28)=-5505302+(x(663)+x(664)+x(665)+x(666)+x(667)+x(668)+x(669));

c(29)=4653696-(x(670)+x(671)+x(672)+x(673)+x(674)+x(675)+x(676));

c(30)=-4961378+(x(670)+x(671)+x(672)+x(673)+x(674)+x(675)+x(676));

c(31)=1997294-(x(677)+x(678)+x(679)+x(680)+x(681)+x(682)+x(683));

c(32)=-2129346+(x(677)+x(678)+x(679)+x(680)+x(681)+x(682)+x(683));

c(33)=58195676-(x(684)+x(685)+x(686)+x(687)+x(688)+x(689)+x(690));

c(34)=-62043324+(x(684)+x(685)+x(686)+x(687)+x(688)+x(689)+x(690));

c(35)=85126888-(x(691)+x(692)+x(693)+x(694)+x(695)+x(696)+x(697));

c(36)=-90755112+(x(691)+x(692)+x(693)+x(694)+x(695)+x(696)+x(697));

c(37)=3889871-(x(698)+x(699)+x(700)+x(701)+x(702)+x(703)+x(704));

c(38)=-4147053+(x(698)+x(699)+x(700)+x(701)+x(702)+x(703)+x(704));

c(39)=22443000-(x(705)+x(706)+x(707)+x(708)+x(709)+x(710)+x(711));

c(40)=-23926834+(x(705)+x(706)+x(707)+x(708)+x(709)+x(710)+x(711));

c(41)=35742722-(x(712)+x(713)+x(714)+x(715)+x(716)+x(717)+x(718));

c(42)=-38105878+(x(712)+x(713)+x(714)+x(715)+x(716)+x(717)+x(718));

c(43)=5707472-(x(719)+x(720)+x(721)+x(722)+x(723)+x(724)+x(725));

c(44)=-6084826+(x(719)+x(720)+x(721)+x(722)+x(723)+x(724)+x(725));

c(45)=40330752-(x(726)+x(727)+x(728)+x(729)+x(730)+x(731)+x(732));

c(46)=-42997248+(x(726)+x(727)+x(728)+x(729)+x(730)+x(731)+x(732));

c(47)=40324654-(x(733)+x(734)+x(735)+x(736)+x(737)+x(738)+x(739));

c(48)=-42990746+(x(733)+x(734)+x(735)+x(736)+x(737)+x(738)+x(739));

c(49)=10028480-(x(740)+x(741)+x(742)+x(743)+x(744)+x(745)+x(746));

c(50)=-10691520+(x(740)+x(741)+x(742)+x(743)+x(744)+x(745)+x(746));

c(51)=3097913-(x(747)+x(748)+x(749)+x(750)+x(751)+x(752)+x(753));

c(52)=-3302733+(x(747)+x(748)+x(749)+x(750)+x(751)+x(752)+x(753));

c(53)=1933704-(x(754)+x(755)+x(756)+x(757)+x(758)+x(759));

c(54)=-2061552+(x(754)+x(755)+x(756)+x(757)+x(758)+x(759));

c(55)=1282351-(x(760)+x(761)+x(762)+x(763)+x(764)+x(765)+x(766));

c(56)=-1367135+(x(760)+x(761)+x(762)+x(763)+x(764)+x(765)+x(766));

c(57)=94206728-(x(767)+x(768)+x(769)+x(770)+x(771)+x(772)+x(773));

c(58)=-100435272+(x(767)+x(768)+x(769)+x(770)+x(771)+x(772)+x(773));

c(59)=11390456-(x(774)+x(775)+x(776)+x(777)+x(778)+x(779)+x(780));

c(60)=-12143544+(x(774)+x(775)+x(776)+x(777)+x(778)+x(779)+x(780));

c(61)=11410784-(x(781)+x(782)+x(783)+x(784)+x(785)+x(786)+x(787));

c(62)=-12165216+(x(781)+x(782)+x(783)+x(784)+x(785)+x(786)+x(787));

c(63)=23785793-(x(788)+x(789)+x(790)+x(791)+x(792)+x(793)+x(794));

c(64)=-25358407+(x(788)+x(789)+x(790)+x(791)+x(792)+x(793)+x(794));

c(65)=40061745-(x(795)+x(796)+x(797)+x(798)+x(799)+x(800)+x(801));

c(66)=-42710455+(x(795)+x(796)+x(797)+x(798)+x(799)+x(800)+x(801));

c(67)=15031201-(x(802)+x(803)+x(804)+x(805)+x(806)+x(807)+x(808));

c(68)=-16024999+(x(802)+x(803)+x(804)+x(805)+x(806)+x(807)+x(808));

c(69)=2360718-(x(809)+x(810)+x(811)+x(812)+x(813)+x(814)+x(815));

c(70)=-2516798+(x(809)+x(810)+x(811)+x(812)+x(813)+x(814)+x(815));

c(71)=3373770-(x(816)+x(817)+x(818)+x(819)+x(820)+x(821)+x(822));

c(72)=-3596830+(x(816)+x(817)+x(818)+x(819)+x(820)+x(821)+x(822));

c(73)=94910077-(x(823)+x(824)+x(825)+x(826)+x(827)+x(828)+x(829));

c(74)=-101185123+(x(823)+x(824)+x(825)+x(826)+x(827)+x(828)+x(829));

c(75)=16000169-(x(830)+x(831)+x(832)+x(833)+x(834)+x(835)+x(836));

c(76)=-17058031+(x(830)+x(831)+x(832)+x(833)+x(834)+x(835)+x(836));

旧的碳约束

ceq(210)=x(1)*1332.5130161269-x(419);

ceq(211)=x(2)*2438.0787824368-x(420);

ceq(212)=x(3)*900.0026726534-x(421);

ceq(213)=x(4)*1165.4680131780-x(422);

ceq(214)=x(5)*543.6181820837-x(423);

ceq(215)=x(6)*255.0949803464-x(424);

ceq(216)=x(7)*108.9463610934-x(425);

ceq(217)=x(8)*2822.3126666153-x(426);

ceq(218)=x(9)*1373.3966621225-x(427);

ceq(219)=x(10)*1067.6588545541-x(428);

ceq(220)=x(11)*478.3647477890-x(429);

ceq(221)=x(12)*558.3929517247-x(430);

ceq(222)=x(13)*205.5395060171-x(431);

ceq(223)=x(14)*923.7503371175-x(432);

ceq(224)=x(15)*1723.3159584115-x(433);

ceq(225)=x(16)*1797.3682186367-x(434);

ceq(226)=x(17)*1363.0532769131-x(435);

ceq(227)=x(18)*2014.3201942019-x(436);

ceq(228)=x(19)*1412.9233713689-x(437);

ceq(229)=x(20)*459.0099437665-x(438);

ceq(230)=x(21)*2601.4528485083-x(439);

ceq(231)=x(22)*4543.3709178729-x(440);

ceq(232)=x(23)*4810.2825017339-x(441);

ceq(233)=x(24)*4159.4690426402-x(442);

ceq(234)=x(25)*5200.0009681175-x(443);

ceq(235)=x(26)*1524.0511693865-x(444);

ceq(236)=x(27)*647.8458465486-x(445);

ceq(237)=x(28)*1070.0460156307-x(446);

ceq(238)=x(29)*2206.3435677958-x(447);

ceq(239)=x(30)*2956.0010092644-x(448);

ceq(240)=x(31)*1081.4795696149-x(449);

ceq(241)=x(32)*823.8480050243-x(450);

ceq(242)=x(33)*1025.8279216730-x(451);

ceq(243)=x(34)*466.7257689577-x(452);

ceq(244)=x(35)*1851.7309400487-x(453);

ceq(245)=x(36)*4699.3775184288-x(454);

ceq(246)=x(37)*4376.4455824415-x(455);

ceq(247)=x(38)*1489.9935476616-x(456);

ceq(248)=x(39)*1555.9885992005-x(457);

ceq(249)=x(40)*498.1667728125-x(458);

ceq(250)=x(41)*69.8549611355-x(459);

ceq(251)=x(42)*218.9031368291-x(460);

ceq(252)=x(43)*25847.5115280649-x(461);

ceq(253)=x(44)*2617.8475878139-x(462);

ceq(254)=x(45)*1125.9116070644-x(463);

ceq(255)=x(46)*1968.1088525836-x(464);

ceq(256)=x(47)*211.7926771279-x(465);

ceq(257)=x(48)*70.9371063088-x(466);

ceq(258)=x(49)*667.8346180799-x(467);

ceq(259)=x(50)*9208.5069063802-x(468);

ceq(260)=x(51)*540114.9425287360-x(469);

ceq(261)=x(52)*2096.4833183048-x(470);

ceq(262)=x(53)*2521.9764613608-x(471);

ceq(263)=x(54)*261.1507758780-x(472);

ceq(264)=x(55)*1603.7252080128-x(473);

ceq(265)=x(56)*63987.7143588431-x(474);

ceq(266)=x(57)*2938.2969491229-x(475);

ceq(267)=x(58)*19672.2056265459-x(476);

ceq(268)=x(59)*3919.0562295060-x(477);

ceq(269)=x(60)*2657.7050176343-x(478);

ceq(270)=x(61)*1440.1540603157-x(479);

ceq(271)=x(62)*4368.0023341762-x(480);

ceq(272)=x(63)*3889.6756291264-x(481);

ceq(273)=x(64)*3287.2023964846-x(482);

ceq(274)=x(65)*3297.7602149292-x(483);

ceq(275)=x(66)*6415.1211757816-x(484);

ceq(276)=x(67)*1197.4124950901-x(485);

ceq(277)=x(68)*3686.7414978921-x(486);

ceq(278)=x(69)*1432.3952631666-x(487);

ceq(279)=x(70)*1298.9265910363-x(488);

ceq(280)=x(71)*7492.1723012423-x(489);

ceq(281)=x(72)*14128.7608903805-x(490);

ceq(282)=x(73)*7625.7774883447-x(491);

ceq(283)=x(74)*16598.8048100249-x(492);

ceq(284)=x(75)*5852.3605653458-x(493);

ceq(285)=x(76)*793.3597224506-x(494);

ceq(286)=x(77)*1530.4930022822-x(495);

ceq(287)=x(78)*3212.3331405263-x(496);

ceq(288)=x(79)*26540.4477179625-x(497);

ceq(289)=x(80)*16048.8770430761-x(498);

ceq(290)=x(81)*1141.9269870164-x(499);

ceq(291)=x(82)*815.9087835255-x(500);

ceq(292)=x(83)*86.7003710197-x(501);

ceq(293)=x(84)*1540.9727947238-x(502);

ceq(294)=x(85)*2847.6470160381-x(503);

ceq(295)=x(86)*2307.4113578332-x(504);

ceq(296)=x(87)*662.1356610013-x(505);

ceq(297)=x(88)*332.3701727485-x(506);

ceq(298)=x(89)*1118.7138936394-x(507);

ceq(299)=x(90)*729.3180751364-x(508);

ceq(300)=x(91)*3348.9012208657-x(509);

ceq(301)=x(92)*7627.8039241433-x(510);

ceq(302)=x(93)*7041.0401946708-x(511);

ceq(303)=x(94)*6483.7823317857-x(512);

ceq(304)=x(95)*2856.0481235738-x(513);

ceq(305)=x(96)*11384.6785762548-x(514);

ceq(306)=x(97)*4392.5526689705-x(515);

ceq(307)=x(98)*5620.2430573360-x(516);

ceq(308)=x(99)*1892.2742602158-x(517);

ceq(309)=x(100)*5670.5386588909-x(518);

ceq(310)=x(101)*1273.1844471651-x(519);

ceq(311)=x(102)*1049.7272845033-x(520);

ceq(312)=x(103)*535.8562969375-x(521);

ceq(313)=x(104)*393.6501855063-x(522);

ceq(314)=x(105)*373.5823707230-x(523);

ceq(315)=x(106)*5546.4141456958-x(524);

ceq(316)=x(107)*10503.7063871682-x(525);

ceq(317)=x(108)*10754.2898735629-x(526);

ceq(318)=x(109)*8451.3076397887-x(527);

ceq(319)=x(110)*3662.7573773488-x(528);

ceq(320)=x(111)*1224.5479993723-x(529);

ceq(321)=x(112)*2347.5567992799-x(530);

ceq(322)=x(113)*3593.4110960003-x(531);

ceq(323)=x(114)*8879.5248339725-x(532);

ceq(324)=x(115)*24644.1622451103-x(533);

ceq(325)=x(116)*1486.8094102427-x(534);

ceq(326)=x(117)*4645.9479587804-x(535);

ceq(327)=x(118)*2300.1263872297-x(536);

ceq(328)=x(119)*1221.8002049798-x(537);

ceq(329)=x(120)*489.0912909332-x(538);

ceq(330)=x(121)*1781.0549731519-x(539);

ceq(331)=x(122)*2073.7963244990-x(540);

ceq(332)=x(123)*122.6719911386-x(541);

ceq(333)=x(124)*866.1900048331-x(542);

ceq(334)=x(125)*3451.5269649575-x(543);

ceq(335)=x(126)*383.4581204954-x(544);

ceq(336)=x(127)*5266.0676327630-x(545);

ceq(337)=x(128)*5420.0087458143-x(546);

ceq(338)=x(129)*6353.8466970307-x(547);

ceq(339)=x(130)*7765.0542345228-x(548);

ceq(340)=x(131)*3075.4890242230-x(549);

ceq(341)=x(342)*14310.4474381864-x(550);

ceq(342)=x(133)*41268.0518204548-x(551);

ceq(343)=x(134)*23944.5971826658-x(552);

ceq(344)=x(135)*7128.4811607770-x(553);

ceq(345)=x(136)*1338.0835123382-x(554);

ceq(346)=x(137)*31357.9345905196-x(555);

ceq(347)=x(138)*6478.4584654860-x(556);

ceq(348)=x(139)*11712.5007298418-x(557);

ceq(349)=x(140)*1760.8222305289-x(558);

ceq(350)=x(141)*5641.2472507838-x(559);

ceq(351)=x(142)*2059.6565119510-x(560);

ceq(352)=x(143)*2954.2982959764-x(561);

ceq(353)=x(144)*3145.7334824168-x(562);

ceq(354)=x(145)*855.4726643421-x(563);

ceq(355)=x(146)*1384.4964958077-x(564);

ceq(356)=x(147)*21586.4954219516-x(565);

ceq(357)=x(148)*11435.4966318719-x(566);

ceq(358)=x(149)*93390.1181615415-x(567);

ceq(359)=x(150)*8747.2321443002-x(568);

ceq(360)=x(151)*548.8811493557-x(569);

ceq(361)=x(152)*536.9432549391-x(570);

ceq(362)=x(153)*944.2432391403-x(571);

ceq(363)=x(154)*1505.6791863563-x(572);

ceq(364)=x(155)*12164.1142071785-x(573);

ceq(365)=x(156)*2146.3030953212-x(574);

ceq(366)=x(157)*1546.3140433935-x(575);

ceq(367)=x(158)*1630.0457239910-x(576);

ceq(368)=x(159)*4098.5749797307-x(577);

ceq(369)=x(160)*7410.5841439578-x(578);

ceq(370)=x(161)*8635.1618193380-x(579);

ceq(371)=x(162)*24494.4971054138-x(580);

ceq(372)=x(163)*2622.2294748361-x(581);

ceq(373)=x(164)*2427.6707675738-x(582);

ceq(374)=x(165)*1774.4648602407-x(583);

ceq(375)=x(166)*601.1019294096-x(584);

ceq(376)=x(167)*747.9065003916-x(585);

ceq(377)=x(168)*7539.1057039027-x(586);

ceq(378)=x(169)*13849.5360119167-x(587);

ceq(379)=x(170)*10566.2770944097-x(588);

ceq(380)=x(171)*5392.6866532898-x(589);

ceq(381)=x(172)*5822.2144212240-x(590);

ceq(382)=x(173)*669.4781666188-x(591);

ceq(383)=x(174)*5188.6152299573-x(592);

ceq(384)=x(175)*2769.9706198388-x(593);

ceq(385)=x(176)*2245.0226263199-x(594);

ceq(386)=x(177)*3376.6116966045-x(595);

ceq(387)=x(178)*946.6276787904-x(596);

ceq(388)=x(179)*1583.9903711973-x(597);

ceq(389)=x(180)*353.7411051652-x(598);

ceq(390)=x(181)*1255.3979813466-x(599);

ceq(391)=x(182)*7219.2483557705-x(600);

ceq(392)=x(183)*2468.0267447145-x(601);

ceq(393)=x(184)*2177.0534687228-x(602);

ceq(394)=x(185)*5688.7593499194-x(603);

ceq(395)=x(186)*1055.3820385694-x(604);

ceq(396)=x(187)*10107.3283094550-x(605);

ceq(397)=x(188)*199592.3537914350-x(606);

ceq(398)=x(189)*2450.1037540087-x(607);

ceq(399)=x(190)*8669.5397029158-x(608);

ceq(400)=x(191)*2299.0196078431-x(609);

ceq(401)=x(192)*7867.5865999105-x(610);

ceq(402)=x(193)*1252.3310023310-x(611);

ceq(403)=x(194)*166.1129568106-x(612);

ceq(404)=x(195)*1030.0734618916-x(613);

ceq(405)=x(196)*3481.0241569195-x(614);

ceq(406)=x(197)*3554.5096393687-x(615);

ceq(407)=x(198)*2036.0365816728-x(616);

ceq(408)=x(199)*2164.5554852488-x(617);

ceq(409)=x(200)*1158.0478827207-x(618);

ceq(410)=x(201)*905.7761358928-x(619);

ceq(411)=x(202)*1361.4212593739-x(620);

ceq(412)=x(203)*5483.3643653926-x(621);

ceq(413)=x(204)*2477.2714097379-x(622);

ceq(414)=x(205)*767.7714924827-x(623);

ceq(415)=x(206)*1837.8473701578-x(624);

ceq(416)=x(207)*938.5609306725-x(625);

ceq(417)=x(208)*126.9021051317-x(626);

ceq(418)=x(209)*5283.9065578133-x(627);

去掉的冗余产出约束：

c(1)=-544283.5271+(x(1)+x(2)+x(3)+x(4)+x(5)+x(6)+x(7)+x(8)+x(9)+x(10)+x(11)+x(12)+x(13)+x(14)+x(15)+x(16)+x(17)+x(18)+x(19)+x(20)+x(21)+x(22)+x(23)+x(24)+x(25)+x(26)+x(27)+x(28)+x(29)+x(30)+x(31)+x(32)+x(33)+x(34)+x(35)+x(36)+x(37)+x(38)+x(39)+x(40)+x(41)+x(42)+x(43)+x(44)+x(45)+x(46)+x(47)+x(48)+x(49)+x(50)+x(51)+x(52)+x(53)+x(54)+x(55)+x(56)+x(57)+x(58)+x(59)+x(60)+x(61)+x(62)+x(63)+x(64)+x(65)+x(66)+x(67)+x(68)+x(69)+x(70)+x(71)+x(72)+x(73)+x(74)+x(75)+x(76)+x(77)+x(78)+x(79)+x(80)+x(81)+x(82)+x(83)+x(84)+x(85)+x(86)+x(87)+x(88)+x(89)+x(90)+x(91)+x(92)+x(93)+x(94)+x(95)+x(96)+x(97)+x(98)+x(99)+x(100)+x(101)+x(102)+x(103)+x(104)+x(105)+x(106)+x(107)+x(108)+x(109)+x(110)+x(111)+x(112)+x(113)+x(114)+x(115)+x(116)+x(117)+x(118)+x(119)+x(120)+x(121)+x(122)+x(123)+x(124)+x(125)+x(126)+x(127)+x(128)+x(129)+x(130)+x(131)+x(132)+x(133)+x(134)+x(135)+x(136)+x(137)+x(138)+x(139)+x(140)+x(141)+x(142)+x(143)+x(144)+x(145)+x(146)+x(147)+x(148)+x(149)+x(150)+x(151)+x(152)+x(153)+x(154)+x(155)+x(156)+x(157)+x(158)+x(159)+x(160)+x(161)+x(162)+x(163)+x(164)+x(165)+x(166)+x(167)+x(168)+x(169)+x(170)+x(171)+x(172)+x(173)+x(174)+x(175)+x(176)+x(177)+x(178)+x(179)+x(180)+x(181)+x(182)+x(183)+x(184)+x(185)+x(186)+x(187)+x(188)+x(189)+x(190)+x(191)+x(192)+x(193)+x(194)+x(195)+x(196)+x(197)+x(198)+x(199)+x(200)+x(201)+x(202)+x(203)+x(204)+x(205)+x(206)+x(207)+x(208)+x(209));

c(1)=285918.0805-(x(1)+x(2)+x(3)+x(4)+x(5)+x(6)+x(7)+x(8)+x(9)+x(10)+x(11)+x(12)+x(13)+x(14)+x(15)+x(16)+x(17)+x(18)+x(19)+x(20)+x(21)+x(22)+x(23)+x(24)+x(25)+x(26)+x(27)+x(28)+x(29)+x(30)+x(31)+x(32)+x(33)+x(34)+x(35)+x(36)+x(37)+x(38)+x(39)+x(40)+x(41)+x(42)+x(43)+x(44)+x(45)+x(46)+x(47)+x(48)+x(49)+x(50)+x(51)+x(52)+x(53)+x(54)+x(55)+x(56)+x(57)+x(58)+x(59)+x(60)+x(61)+x(62)+x(63)+x(64)+x(65)+x(66)+x(67)+x(68)+x(69)+x(70)+x(71)+x(72)+x(73)+x(74)+x(75)+x(76)+x(77)+x(78)+x(79)+x(80)+x(81)+x(82)+x(83)+x(84)+x(85)+x(86)+x(87)+x(88)+x(89)+x(90)+x(91)+x(92)+x(93)+x(94)+x(95)+x(96)+x(97)+x(98)+x(99)+x(100)+x(101)+x(102)+x(103)+x(104)+x(105)+x(106)+x(107)+x(108)+x(109)+x(110)+x(111)+x(112)+x(113)+x(114)+x(115)+x(116)+x(117)+x(118)+x(119)+x(120)+x(121)+x(122)+x(123)+x(124)+x(125)+x(126)+x(127)+x(128)+x(129)+x(130)+x(131)+x(132)+x(133)+x(134)+x(135)+x(136)+x(137)+x(138)+x(139)+x(140)+x(141)+x(142)+x(143)+x(144)+x(145)+x(146)+x(147)+x(148)+x(149)+x(150)+x(151)+x(152)+x(153)+x(154)+x(155)+x(156)+x(157)+x(158)+x(159)+x(160)+x(161)+x(162)+x(163)+x(164)+x(165)+x(166)+x(167)+x(168)+x(169)+x(170)+x(171)+x(172)+x(173)+x(174)+x(175)+x(176)+x(177)+x(178)+x(179)+x(180)+x(181)+x(182)+x(183)+x(184)+x(185)+x(186)+x(187)+x(188)+x(189)+x(190)+x(191)+x(192)+x(193)+x(194)+x(195)+x(196)+x(197)+x(198)+x(199)+x(200)+x(201)+x(202)+x(203)+x(204)+x(205)+x(206)+x(207)+x(208)+x(209));
